# Wide-range screening of anti-inflammatory compounds in tomato using LC-MS and elucidating the mechanism of their functions

**DOI:** 10.1371/journal.pone.0191203

**Published:** 2018-01-12

**Authors:** Shinsuke Mohri, Haruya Takahashi, Maiko Sakai, Shingo Takahashi, Naoko Waki, Koichi Aizawa, Hiroyuki Suganuma, Takeshi Ara, Yasuki Matsumura, Daisuke Shibata, Tsuyoshi Goto, Teruo Kawada

**Affiliations:** 1 Laboratory of Molecular Function of Food, Graduate School of Agriculture, Kyoto University, Uji, Kyoto, Japan; 2 KAGOME Tomato Discoveries Laboratory, Graduate School of Agriculture, Kyoto University, Kyoto, Japan; 3 Innovation Division, KAGOME CO., LTD., Tochigi, Japan; 4 Laboratory of Quality Analysis and Assessment, Graduate School of Agriculture, Kyoto University, Uji, Kyoto, Japan; 5 Kazusa DNA Research Institutes, Kazusa-Kamatari, Kisarazu, Chiba, Japan; 6 Research Unit for Physiological Chemistry, Kyoto University, Kyoto, Japan; Tokyo University of Agriculture, JAPAN

## Abstract

Obesity-induced chronic inflammation is a key factor in type 2 diabetes. A vicious cycle involving pro-inflammatory mediators between adipocytes and macrophages is a common cause of chronic inflammation in the adipose tissue. Tomato is one of the most popular vegetables and is associated with a reduced risk of diabetes. However, the molecular mechanism underlying the effect of tomato on diabetes is unclear. In this study, we focused on anti-inflammatory compounds in tomato. We found that the extract of tomato reduced plasma glucose and inflammatory markers in mice. We screened anti-inflammatory fractions in tomato using lipopolysaccharide-stimulated RAW264.7 macrophages, and active compounds were estimated by liquid chromatography-mass spectrometry over a wide range. Surprisingly, a large number of compounds including oxylipin and coumarin derivatives were estimated as anti-inflammatory compounds. Especially, 9-oxo-octadecadienoic acid and daphnetin suppressed pro-inflammatory cytokines in RAW264.7 macrophages inhibiting mitogen-activated protein kinase phosphorylation and inhibitor of kappa B α protein degradation. These findings suggest that tomato containing diverse anti-inflammatory compounds ameliorates chronic inflammation in obese adipose tissue.

## Introduction

Obesity is a major risk factor for the development of numerous complications, including type 2 diabetes and cardiovascular diseases[1,2]. Lifestyle-related diseases result from abnormal glucose and lipid metabolism, which are primarily caused by obesity[2]. Obesity is an excessive accumulation of adipose tissue that has become a worldwide concern. In recent years, several studies have reported that obesity has been closely associated with low-grade chronic inflammation in the adipose tissue[3,4–6]. The inflammation state of obesity increases infiltration of macrophages in the adipose tissue that are recruited by the monocyte chemoattractant protein (MCP)-1 released from hypertrophied adipocytes[7–9]. These infiltrating macrophages cause the secretion of various inflammatory cytokines such as nitric oxide (NO) and tumor necrosis factor (TNF)-α, which can cause insulin resistance[10,11]. The interaction between adipocytes and infiltrating macrophages in the adipose tissue contributes to the vicious cycle that leads to chronic inflammation and glucose metabolism disorder under conditions of obesity[12,13]. Thus, in order to improve glucose metabolism, it is important to inhibit the production of these inflammatory cytokines and suppress the low-grade inflammation in obese adipose tissue.

Tomato is one of the most popular and commonly consumed fresh vegetables in the world. Previous studies have indicated that the dietary intake of tomato is linked to a reduced risk of chronic diseases such as cardiovascular diseases and type 2 diabetes[14–16]. Furthermore, tomato consumption reduces inflammation by decreasing inflammatory cytokines in overweight and obese humans[17,18]. These interesting effects of tomato consumption have been elucidated, but the active compounds in tomato are not fully understood. In recent years, we demonstrated that tomato contained fatty acid derivatives (oxylipin) that activated lipid metabolism via peroxisome proliferator-activated receptor (PPAR) α activation[19–21], which is important for fatty acid oxidation[22–24]. Although the effect of compounds in tomato on lipid metabolism has been elucidated, little is known about the effect on glucose metabolism.

The aim of this study was to identify anti-inflammatory compounds in tomato and to show their mechanisms of action. In the present study, we demonstrated that tomato extract had the ability to reduce plasma glucose level in mice. We focused on anti-inflammatory compounds with the potential to reduce inflammatory cytokines associated with glucose metabolism disorder in obesity. To unravel the active compounds in tomato, we screened anti-inflammatory fractions of tomato extract by measuring NO production in lipopolysaccharide (LPS)-stimulated RAW264.7 macrophages. In addition, we attempted to identify active compounds from the anti-inflammatory fractions using liquid chromatography-mass spectrometry (LC-MS). This wide-range screening revealed that tomato contained a large number of anti-inflammatory fractions and diverse anti-inflammatory compounds, including oxylipin and coumarin. Representative compound of oxylipin and coumarin derivatives (9-oxo-ctadecadienoic acid (9-oxo-ODA) and daphnetin (7,8-dihydroxycoumarin)) inhibited inflammatory cytokines by suppressing mitogen-activated protein kinase (MAPK) phosphorylation and inhibitor kappa B (IκB)-α protein degradation in RAW264.7 macrophages. Moreover, 9-oxo-ODA was detected *in vivo* and tended to increase in the white adipose tissue (WAT) under tomato extract treatment. These findings indicated that various anti-inflammatory compounds in tomato inhibited chronic inflammation between adipocytes and macrophages in obese adipose tissue, suggesting that tomato might be a valuable food to ameliorate glucose metabolism disorder under conditions of obesity.

## Materials and methods

### Plant materials and chemicals

In this study, we used tomatoes that were provided by KAGOME CO., LTD. (Nasushiobara, Tochigi, Japan) (identifier No. *KTP001*). All the other chemicals used were from Invitrogen Corp. (Carlsbad, CA, USA), Nacalai Tesque Inc. (Kyoto, Japan), or Wako (Osaka, Japan) and were guaranteed to be of reagent-, high-performance liquid chromatography (HPLC)-, or tissue culture-grade.

### Animal experiments

Mice were kept in individual cages in a temperature-controlled room at 23 ± 1°C and maintained under a constant 12 h light/dark cycle. Male C57BL/6 mice were purchased from CLEA Japan (Tokyo, Japan). 7-week-old mice were maintained for 7 days on a normal diet (ND) (Research Diet Inc., New Brunswick, NJ, USA) and then divided into two groups of similar average body weight. Each group was maintained on a high-fat diet (HFD) containing 60% kcal fat (Research Diet Inc.) or HFD containing 1% tomato extract (section *2*.*3*) for 10 weeks. This animal experiment was performed under free-feeding conditions. At the end of the treatment period, anesthetized mice were sacrificed by cervical dislocation, and blood and organ samples were collected. Plasma triglyceride (TG), glucose, and non-esterified fatty acid (NEFA) levels were measured using the TG E-test Wako kit (Wako), Glucose CII-test (Wako), and NEFA C-test (Wako), respectively. All animal experiments were approved by the Kyoto University Animal Care Committee (approval code: 28–76).

### Extract preparation and fractionation of crude extract

The extraction and fractionation of tomatoes were performed as follows. The components in tomato were extracted from freeze-dried tomato powder using ethanol (EtOH) at room temperature for 24 h. The EtOH extract was partitioned with ethyl acetate (EtOAc) (tomato extract) and water mixture. The large-scale tomato extract for animal experiments was prepared in TOKIWA Phytochemical Co., Ltd. (Chiba, Japan). On the guidance of NO assay (section *2*.*7*), the soluble portion of tomato extract was further partitioned with *n*-hexane (Hexane extract) and 90% methanol (90% MeOH extract) mixture. Each soluble portion was fractionated by silica gel open column chromatography (eluted with Hexane-EtOAc and MeOH, Hexane: EtOAc = 100: 0 (Ⅰ), 75: 25 (II), 50: 50 (III), 25: 75 (IV), 0: 100 (V), and 100% MeOH (Ⅵ)).

After silica gel open column chromatography, the Hexane and 90% MeOH extract (H-III, M-II, III, and IV) was fractionated by reverse-phase HPLC on a 5C_18_-AR-II octa decyl silyl (ODS) column (6.0×150 mm; Nacalai Tesque) using a mobile phase of water (solvent A) and acetonitrile (solvent B) with 0.1% v/v formic acid added to both solvents. The Hexane extract (H-V) was fractionated by reverse-phase HPLC on a 5C_8_-MS ODS column (6.0×150 mm; Nacalai Tesque) using the same mobile phase. The program began with 1% solvent B in solvent A followed by a linear elution gradient from 1 to 100% solvent B in solvent A for 130 min. To monitor HPLC elution, a diode array detector was used in the range of 200–700 nm. Flow rate was set at 1.0 mL/min. Eluted fractions were collected at 1 mL/min. The above-mentioned solvents in extracts and eluted fractions were evaporated under vacuum at 37°C using a rotary evaporator. The effects of evaporated samples re-dissolved in EtOH on the production of the pro-inflammatory mediators were examined.

### mRNA expression levels

Total RNA was prepared using Sepasol (Nacalai Tesque) according to the manufacturer protocol. Using Moloney Murine Leukemia Virus (M-MLV) reverse transcriptase (Life Technologies Japan Ltd., Tokyo, Japan), total RNA was reverse transcribed, in accordance to the manufacturer instructions using a thermal cycler (Takara PCR Thermal Cycler SP; Takara, Shiga, Japan). To quantify mRNA expression levels, real-time quantitative polymerase chain reaction (RT-PCR) analysis was performed with a LightCycler System (Roche Diagnostics, Mannheim, Germany), using SYBR green fluorescence signals as described previously[25]. The oligonucleotide primer sets for mouse *36B4*, *β-Actin*, *Nos2*, and *Mcp-1* genes were designed using a PCR primer selection program at the website of the Virtual Genomic Center from GenBank, and the sequences are shown in Table 1. All mRNA expression data are presented as ratios relative to the control in each experiment.

**Table 1:** Oligonucleotide primers used for mRNA analysis.

Gene	Forward primer	Reverse primer
*Nos2*	GCCTTCAACACCAAGGTTGTC	GCGCAGAACTGAGGGTACAT
*Mcp-1*	GACCCCAAGAAGGAATGGGT	ACCTTAGGGCAGTGCAGTT
*β-Actin*	AACACCCCAGCCATGTACGTAG	TGTCAAAGAAAGGGTGTAAAACGC
*36B4*	TCCTTCTTCCAGGCTTTGGG	GACACCCTCCAGAAAGCGAG

### LC-MS analysis

The compounds of HPLC fractions and white adipose tissue were assessed using a LC-MS system as previously described[21,25]. Briefly, each HPLC fraction was dissolved in 1mL of extraction solvent (99.5% EtOH). Each white adipose tissue was homogenized in 1mL of extraction solvent with mixer, and then the solvent was centrifuged. After centrifugation (15,000 rpm, 10 min, 4°C), the supernatant was collected for use as an extract. The extract was filtered through a 0.2-μm-pore polyvinylidene difluoride (PVDF) membrane (Whatman, Brentford, UK), and the filtrate was used for LC-MS. LC-MS was performed using a Waters Acquity UPLC system (Milford, MA, USA) coupled to a Xevo QTOF-MS equipped with an electrospray ionization source (ESI).

An aliquot of the extracted sample (3 μL) was injected into an Acquity UPLC BEH-C18 reversed-phase column (2.1×100 mm column size; 1.7 μm particle size). Mobile phases A (water and 0.1% formic acid) and B (acetonitrile and 0.1% formic acid) were used. The column temperature was set at 40°C. The buffer gradient consisted of 30% to 50% B for 0–4 min, 50% to 85% B for 4–14 min, 99% B for 14–17 min, and 30% B for 3 min at a flow rate of 300 μL/min. The buffer gradient for daphnetin and esculetin analysis consisted of 1% B for 0–1 min, 1% to 50% B for 1–6 min, 50% to 99% B for 6–6.1 min, 99% B for 6.1–10 min, and 1% B for 5 min. Data were acquired with MassLynx software (Waters, Manchester, UK). External mass calibration was performed following the manufacturer protocol.

### Cell culture

Cell culture was performed as previously described[26]. Briefly, the RAW264.7 macrophage was cultured in Dulbecco’s modified Eagle’s medium (DMEM) with 10% fetal bovine serum (FBS) and 100 U/mL penicillin and 100 μg/mL streptomycin at 37°C under a humidified 5% CO_2_ atmosphere. To measure MCP-1, TNF-α, and NO levels, RAW264.7 cells were treated with 100 ng/mL LPS and tomato extract, fraction, or authentic sample at various concentrations in serum-free medium for 24 h. 3T3-L1 preadipocytes were subcultured in DMEM with 10% FBS supplemented with 100 U/mL penicillin and 100 μg/mL streptomycin at 37°C under a humidified 5% CO_2_ atmosphere. The differentiation of 3T3-L1 preadipocytes was induced using adipogenic agents [0.5 mM 3-isobutyl-1-methylxanthine, 0.25 μM dexamethasone, and 10 μg/mL insulin] in DMEM containing 10% FBS for 2 days after the cells reached confluence (day 0). Then, the medium was replaced with DMEM containing 10% FBS and 5 μg/mL insulin, which was replaced with fresh medium every 2 days. Twenty days after the differentiation induction, the cells that accumulated large lipid droplets were used as hypertrophied 3T3-L1 adipocytes.

In the co-culture system, adipocytes and macrophages were co-cultured in a contact system as previously described[12]. Briefly, RAW264.7 macrophages (1×10^5^ cells/mL) were plated onto dishes with serum-starved and hypertrophied 3T3-L1 cells, and the co-culture was incubated in serum-free DMEM for 24 h. RAW264.7 and 3T3-L1 cells of equal numbers to those in the co-culture were cultured separately as control cultures. 9-oxo-ODA, daphnetin, or tomato extract was added to the co-culture at various concentrations as shown in each figure. After 24 h of treatment, culture supernatants were collected, and inflammatory mediators were measured as described below.

### Measurement of inflammatory mediators

The concentrations of MCP-1 and TNF-α in the culture supernatants were determined by enzyme-linked immunosorbent assay (ELISA) conducted using a Ready-SET-Go mouse MCP-1 and TNF-α kit (eBioscience, San Diego, CA, USA) according to the manufacturer protocol. The amount of NO in the cell-free culture supernatants was measured using Griess reagent[27]. Briefly, 100 μL of supernatant were mixed with an equivalent volume of Griess reagent [1:1 (v/v) of 0.1% *N*-(1-naphthyl)-ethylenediamine in distilled water and 1% sulfanilamide in 5% phosphoric acid] in a 96-well flat-bottom plate. After 10 min, absorbance at 550 nm was measured, and the amount of NO was calculated from the sodium nitrite (NaNO_2_) standard curve.

### Western blotting

Proteins from RAW264.7 macrophages were solubilized in lysis buffer containing 20 mM Tris HCl (pH 7.5), 15 mM NaCl, 1% Triton X100, and a protease and phosphatase inhibitor cocktail (Nacalai Tesque). The protein concentration of the cell lysate was determined using detergent compatible (DC) protein assay (BioRad Laboratories, Hercules, CA, USA). Protein samples were subjected to sodium dodecyl sulfate-polyacrylamide gel electrophoresis (SDS-PAGE), and separated products were transferred to PVDF membrane (Millipore, Bedford, MA, USA). After blocking with 5% skim milk in TBS and 0.1% Tween20, the membrane was incubated with an anti-extracellular signal-regulated kinase (anti-ERK), anti-phosphorylated ERK (anti-pERK), anti-c-Jun N-terminal kinase (anti-JNK), anti-p38, anti-βactin (Cell Signaling Technology, Beverly, MA, USA), or anti-IκB-α (Santa Cruz Biotechnology, Santa Cruz, CA, USA) antibody overnight, and then with a secondary antibody conjugated to horseradish peroxidase (HRP) (Santa Cruz Biotechnology). The secondary antibody was visualized using chemiluminescent HRP substrate (Millipore). For band quantification, ImageJ (National Institutes of Health, Bethesda, MD, USA) was used.

### Statistical analysis

The data are presented as means ± standard error of the mean (SEM). Data were assessed by Student’s *t*-test or one-way ANOVA and Dunnett’s multiple comparison tests. Differences were considered significant at *p* < 0.05.

## Results

### Effects of tomato extract on metabolism in vivo

**Fig 1:**
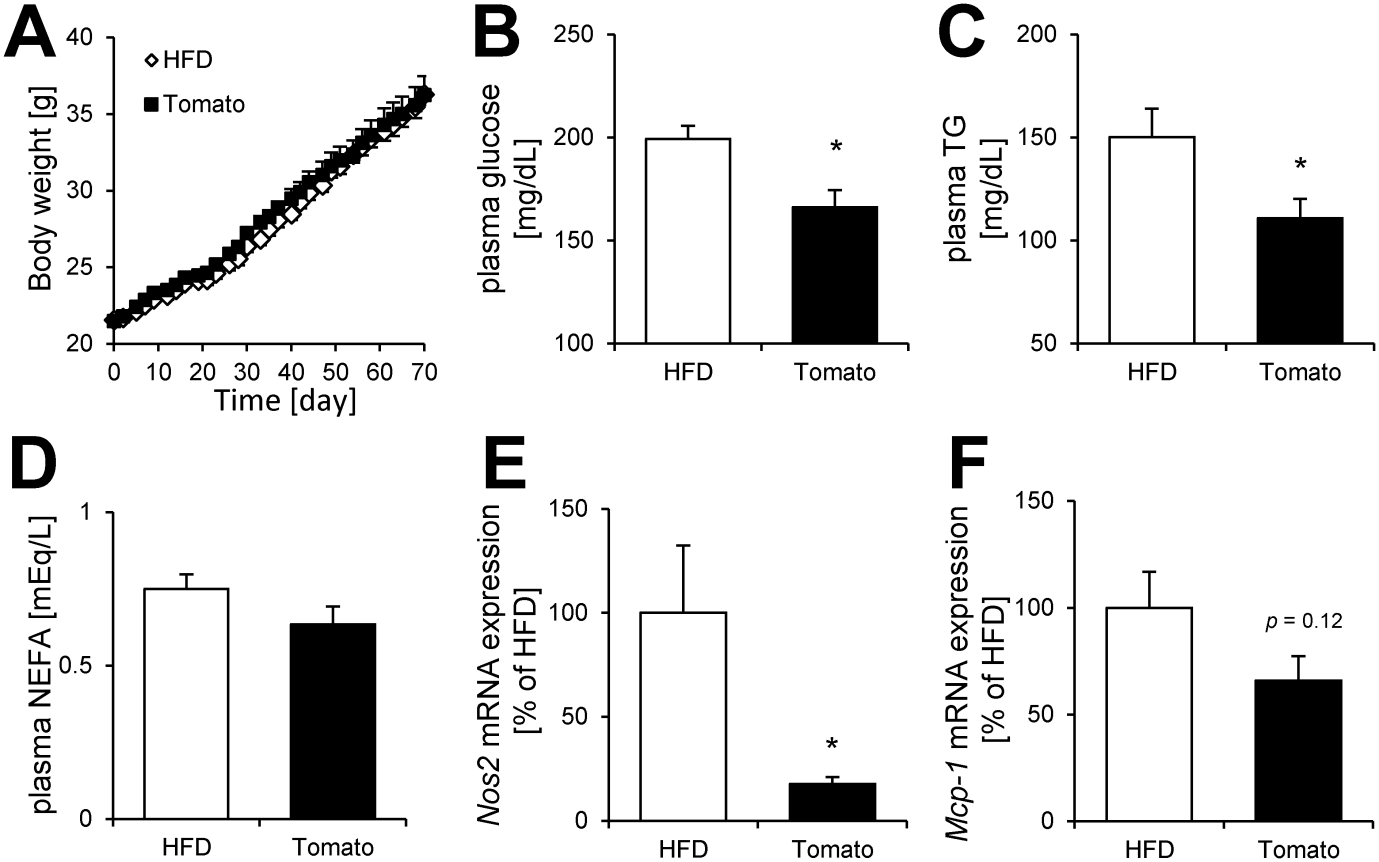
Effect of tomato extract on metabolism in mice. **(A)** Body weight gain, plasma **(B)** glucose, **(C)** TG, and **(D)** NEFA levels in C57BL/6 mice. Effect of tomato extract on **(E)** *Nos2* and **(F)** *Mcp-1* mRNA expression levels in white adipose tissue. Data are presented as the mean ± SEM (n = 8–10/group), *p < 0.05 vs. HFD group. HFD; high fat diet, Tomato; tomato extract.

First, we investigated the effect of tomato extract on metabolism in mice. No significant differences in body weight, tissue weight, and food intake between the HFD group and tomato extract group were observed (Fig 1A and Table 2). Although plasma total NEFA levels did not change (Fig 1D), plasma glucose and TG levels were reduced by tomato extract treatment (Fig 1B and 1C). In the white adipose tissue, we observed that the mRNA expression of *Nos2* involved in NO production was markedly decreased by tomato extract treatment (Fig 1E). Furthermore, the expression of *Mcp-1* tended to decrease under tomato extract treatment (Fig 1F). These results suggested that anti-inflammatory effect of tomato extract improved glucose metabolism disorder in mice.

**Table 2:** Effect of tomato extract on tissue weight in mice.

Tissue weight (g)	HFD	Tomato
WAT	3.76±0.41	3.55±0.36
BAT	0.14±0.01	0.13±0.01
Liver	1.26±0.05	1.26±0.07
Kidney	0.32±0.01	0.33±0.01

### Screening of anti-inflammatory fraction by silica gel column chromatography and HPLC in NO assay

**Fig 2:**
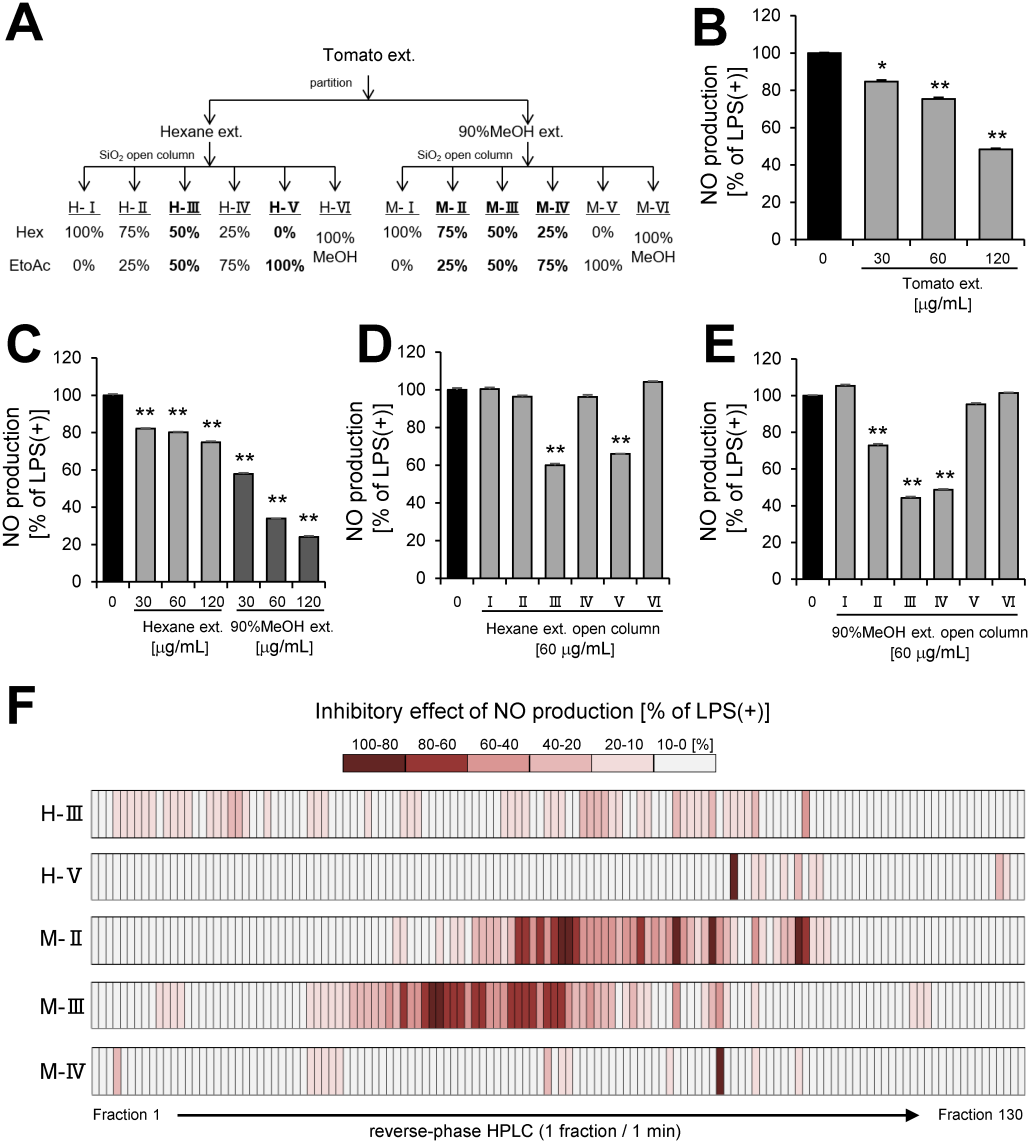
Screening of partitioned fraction in NO assay. **(A)** Scheme of partitioning and open column chromatography of tomato extract. **(B)** NO production by RAW264.7 cell stimulated with LPS (100 ng/mL) and incubated with tomato extract. **(C)** NO production by RAW264 cell stimulated with LPS and incubated with partition fractions (Hexane, 90% MeOH extract) of tomato extract. NO production by RAW264.7 cell stimulated with LPS and incubated with purified fractions of **(D)** Hexane or **(E)** 90% MeOH extract by open column chromatography. **(F)** Heat map of inhibitory effect of NO production by RAW264.7 cell stimulated with LPS and incubated with HPLC fractions. Data are presented as means ± SEM (n = 3/group). *p < 0.05, **p < 0.01 vs. LPS alone.

To determine tomato extract (Fig 2A) has anti-inflammatory effect, we investigated whether tomato extract inhibited NO production in LPS-stimulated RAW 264.7 macrophages. Tomato extract significantly inhibited NO production in a dose-dependent manner (Fig 2B). To identify anti-inflammatory compounds in tomato extract, the extract was partitioned with *n*-hexane and 90% MeOH mixture (Fig 2A), and the inhibitory effects of these portions (Hexane extract and 90% MeOH extract) on NO production by LPS-stimulated RAW264.7 macrophages were examined. The assay revealed that each portion inhibited NO production in a dose-dependent manner (Fig 2C). Therefore, we further fractioned each portion by silica gel open column chromatography (Fig 2A) and obtained five anti-inflammatory fractions (H-III, H-V, M-II, M-III, and M-IV) (Fig 2D and 2E). To purify the active compounds in the five anti-inflammatory fractions of open column chromatography, we separated the fractions by reverse-phase HPLC and obtained 650 HPLC fractions (Fig 2F). NO assay was performed on the 650 fractions acquired by HPLC. In consequence, a lot of anti-inflammatory fractions were presented in HPLC fractions, including 9 fractions suppressed 80–100% of NO production, 21 fractions suppressed 60–80% of NO production, 27 fractions suppressed 40–60% of NO production, 39 fractions suppressed 20–40% of NO production, and 89 fractions suppressed 10–20% of NO production. Moreover, an overview of anti-inflammatory effect of tomato represented by the heat map indicated that 90% MeOH extract contained more potent anti-inflammatory fractions (Fig 2F).

### Identification of anti-inflammatory compounds in HPLC fractions by LC-MS

**Fig 3:**
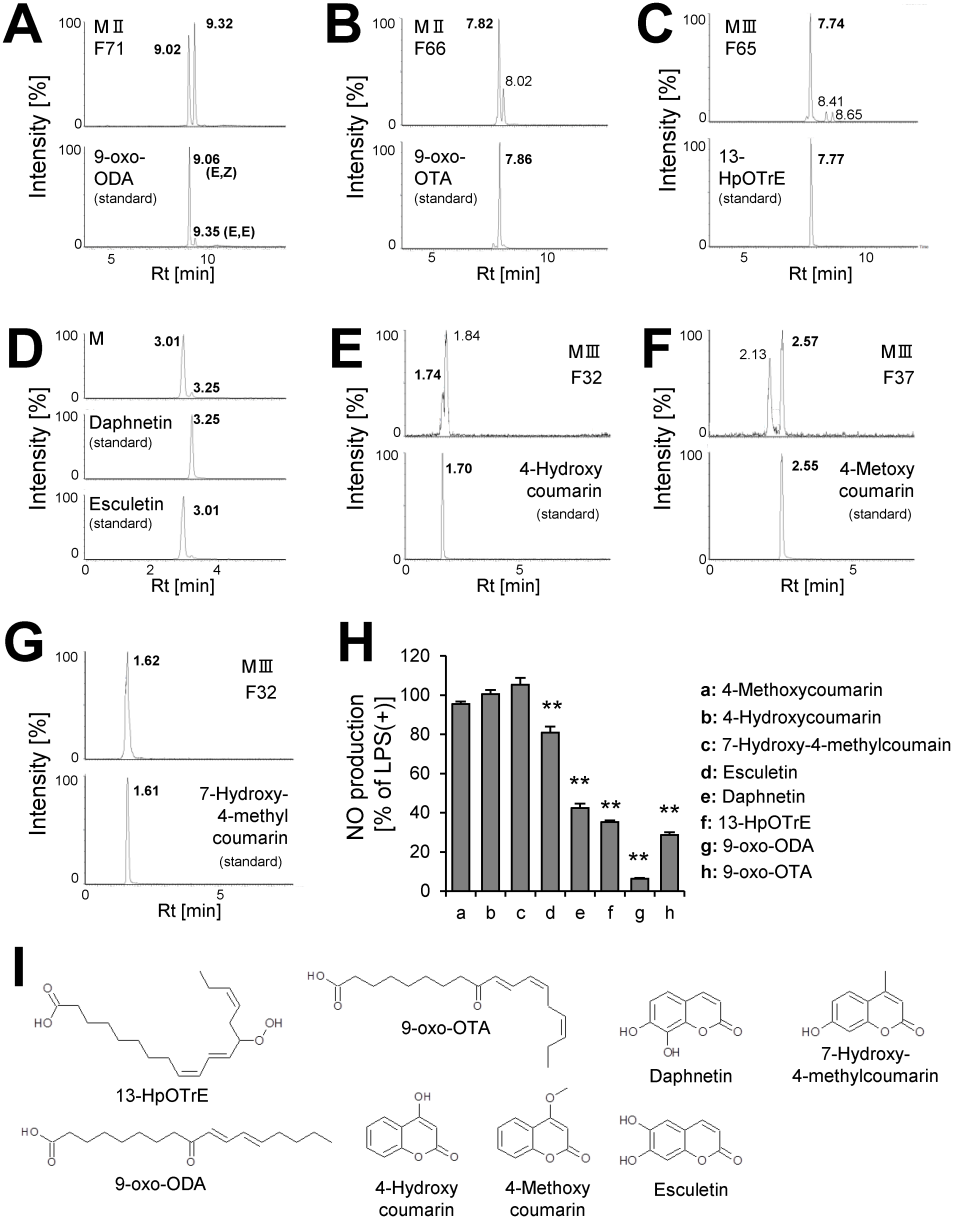
Identification of anti-inflammatory compounds. The extracted ion chromatogram of **(A)** 9-oxo-ODA (m/z = 293.209), **(B)** 9-oxo-OTA (m/z = 291.196), **(C)** 13-HpOTrE (m/z = 309.20), **(D)** daphnetin and esculetin (m/z = 177.020), **(E)** 4-hydroxycoumarin (m/z = 163.036), **(F)** 4-methoxycoumarin (m/z = 177.049), and **(G)** 7-hydroxy-4-methylcoumarin (m/z = 177.049). **(H)** Effect of identified compounds (50 μM) on NO secretion in RAW264.7 cell stimulated with LPS. Data are presented as means ± SEM (n = 3/group). *p < 0.05, **p < 0.01 vs. LPS alone. **(I)** Structure of identified compounds. M; 90% MeOH extract, H; hexane extract, F; HPLC fraction.

To identify the anti-inflammatory compounds in active HPLC fractions, we analyzed these fractions by LC-MS. The results showed that a wide variety of active compounds were estimated in HexaneIII (Table 3), HexaneV(Table 4), 90% MeOHII (Table 5), 90% MeOHIII (Table 6), and 90% MeOHIV (Table 7)-HPLC fractions. Interestingly, we noticed that a number of lipid and coumarin analogs were present in these compounds (Tables 3–7). Therefore, we attempted to identify oxylipin and coumarin analogs in 90% MeOHII, III, and IV extracts containing more potent anti-inflammatory fractions by LC-MS. Consequently, we identified 3 compounds of oxylipin (9-oxo-ODA (Fig 3A), 9-oxo-OTA (Fig 3B), and 13-HpOTrE (Fig 3C)), and 5 compounds of coumarin derivatives (daphnetin (Fig 3D), esculetin (Fig 3D), 4-hydroxycoumarin (Fig 3E), 4-methoxycoumarin (Fig 3F), and 7-hydroxy-4-methylcoumarin (Fig 3G)). At first, the ion chromatogram peak of estimated molecule with the formula C_9_H_6_O_4_ (annotated as daphnetin and esculetin, Table 7) in 90% MeOHIV F4 extract did not separate, and we could not identify the compound relative to this fraction. Therefore, we attempted to separate the peak in the 90% MeOH extract, and consequently both daphnetin and esculetin were included (Fig 3D). Next, the inhibitory effects of these compounds (Fig 3I) on NO production by LPS-stimulated RAW264.7 macrophages were examined. We showed that 9-oxo-ODA and daphnetin significantly inhibited NO production (Fig 3H). Thus, we used 9-oxo-ODA and daphnetin on the behalf of lipid and coumarin derivatives derived from tomato extract to elucidate the potential to suppress the production of pro-inflammatory mediators.

**Table 3:** LC-MS analysis data of Hexane III—HPLC fraction.

HPLC Fraction NO.	Rt (min)	m/z	Ion Form	Estimated Molecule Formula	Annotation
5	n.d.				
13	n.d.				
20	n.d.				
32	n.d.				
44	n.d.				
58	n.d.				
65	7.72	291.190	[M-H]-	C18H28O3	13-oxo-OTA
65	7.82	291.190	[M-H]-	C18H28O3	9-oxo-OTA
65	10.45	n.i.			
70	8.99	293.211	[M-H]-	C18H30O3	9-oxo-(EZ)-ODA
78	11.15	297.243	[M-H]-	C18H34O3	Fatty acid derivative
82	12.13	277.213	[M-H]-	C18H30O2	Linolenic acid
82	12.29	n.i.			
82	13.64	279.231	[M-H]-	C18H32O2	Linoleic acid
87	13.53	n.i.			
87	13.67	279.231	[M-H]-	C18H32O2	Linoleic acid
87	13.84	n.i.			
93	14.95	255.230	[M-H]-	C16H32O2	Palmitic acid
93	15.31	635.290	[M-H]-	C31H40N8O7	?
100	15.95	n.i.			

n.d.; not ditected

n.i.; not identified parent ion

?; not annotated from database

**Table 4:** LC-MS analysis data of Hexane V—HPLC fraction.

HPLC Fraction NO.	Rt (min)	m/z	Ion Form	Estimated Molecule Formula	Annotation
81	11.52	n.i.			
81	11.95	n.i.			
81	12.33	n.i.			
87	12.79	256.263	[M+H]+	C16H33NO	Fatty amide
87	13.29	282.278	[M+H]+	C18H35NO	Fatty amide
87	13.58	n.i.			
93	14.97	255.230	[M-H]-	C16H32O2	Palmitic acid
100	15.96	n.i.			
114	16.08	n.i.			
122	16.29	819.556	[M+H]+	C39H70N12O7	?

n.d.; not ditected

n.i.; not identified parent ion

?; not annotated from database

**Table 5:** LC-MS analysis data of 90%MeOH II—HPLC fraction.

HPLC Fraction NO.	Rt (min)	m/z	Ion Form	Estimated Molecule Formula	Annotation
44	n.d.				
52	n.d.				
55	17.6	537.535	[M+H]+	C34H68N2O2	?
61	n.d.				
63	7.15	n.i.			
66	7.74	n.i.			
66	7.83	291.197	[M-H]-	C18H28O3	9-oxo-OTA
66	8.13	n.i.			
71	9.02	293.209	[M-H]-	C18H30O3	9-oxo-(EZ)-ODA
71	9.32	293.209	[M-H]-	C18H30O3	9-oxo-(EE)-ODA
73	9.46	364.286	[M-H]-	C22H39NO3	?
73	9.69	364.286	[M-H]-	C22H39NO3	?
73	9.74	293.212	[M-H]-	C18H30O3	Oxylipin
73	9.98	364.286	[M-H]-	C22H39NO3	?
77	9.68	364.286	[M-H]-	C22H39NO3	?
82	12.16	277.216	[M-H]-	C18H30O2	Linolenic acid
87	13.60	n.i.			
87	13.64	279.230	[M-H]-	C18H32O2	Linoleic acid
93	14.97	253.228	[M-H]-	C16H32O2	Palmitic acid
99	15.88	277.142	[M-H]-	C16H22O4	?

n.d.; not ditected

n.i.; not identified parent ion

?; not annotated from database

**Table 6:** LC-MS analysis data of 90%MeOH III—HPLC fraction.

HPLC Fraction NO.	Rt (min)	m/z	Ion Form	Estimated Molecule Formula	Annotation
11	n.d.				
32	1.62	314.139	[M+H]+	C18H19NO4	?
32	1.62	177.054	[M+H]+	C10H8O3	7-Hydroxy-4-methylcoumarin
32	1.70	163.036	[M+H]+	C9H6O3	4-Hydroxycoumarin
32	1.86	289.072	[M+H]+	C15H12O6	Eriodictyol
37	2.13	177.049	[M+H]+	C5H8N2O5	?
37	2.44	273.073	[M+H]+	C15H12O5	Naringenin
37	2.57	177.049	[M+H]+	C10H8O3	4-Metoxycoumarin
44	3.58	181.0636	[M+H]+	C13H8O	9-Fluorenone
49	4.15	195.139	[M+H]+	C12H18O2	Fatty acid derivative
49	4.39	339.181	[M-H]-	C18H28O6	Fatty acid derivative
49	5.67	230.249	[M+H]+	C14H31NO	Xestoaminol C
52	4.75	355.248	[M-H]-	C20H36O5	Fatty acid derivative
52	4.86	355.248	[M-H]-	C20H36O5	Fatty acid derivative
54	5.11	357.262	[M-H]-	C20H38O5	Fatty acid derivative
54	5.18	n.i.			
54	5.45	329.233	[M-H]-	C18H34O5	Oxylipin
54	5.45	445.150	[M-H]-	C23H26O9	Flavonoid
61	6.66	311.222	[M-H]-	C18H32O4	Oxylipin
61	6.82	357.262	[M-H]-	C20H38O5	Fatty acid derivative
61	6.89	309.207	[M-H]-	C18H30O4	Oxylipin
65	7.74	333.205	[M+H]+	C20H28O4	Fatty acid derivative
65	7.74	309.202	[M-H]-	C18H30O4	13-HpOTrE
65	7.82	291.196	[M-H]-	C18H28O3	9-oxo-OTA
70	9.02	293.208	[M-H]-	C18H30O3	9-oxo-(EZ)-ODA
70	9.32	293.208	[M-H]-	C18H30O3	9-oxo-(EE)-ODA
76	10.48	261.224	[M+H]+	C18H28O	Fatty acid derivative
76	10.48	225.223	[M+H]+	C15H28O	Fatty acid derivative
76	10.82	261.224	[M+H]+	C18H28O	Fatty acid derivative
82	12.32	263.238	[M+H]+	C18H30O	Isoprenoid
88	13.83	429.286	[M-H]-	C23H42O7	?
88	14.12	437.291	[M-H]-	C25H42O6	Isoprenoid
93	15.00	253.232	[M-H]-	C16H32O2	Palmitic acid

n.d.; not ditected

n.i.; not identified parent ion

?; not annotated from database

**Table 7:** LC-MS analysis data of 90%MeOH IV—HPLC fraction.

HPLC Fraction NO.	Rt (min)	m/z	Ion Form	Estimated Molecule Formula	Annotation
4	0.78	416.228	[M+H]+	C20H33NO8	?
4	0.78	341.088	[M-H]-	C15H18O9	Caffeic acid glucoside
4	0.78	175.024	[M-H]-	C6H8O6	Ascorbate
4	0.97	177.042	[M-H]-	C9H6O4	Daphnetin or Esculetin
64	7.28	200.202	[M+H]+	C12H25NO	Fatty amide
64	7.46	265.148	[M-H]-	C15H22O4	Terpenoid
64	7.62	n.i.			
64	7.82	n.i.			
81	11.49	658.4413	[M+H]+	C35H64NO8P	Lysophospholipid
81	11.88	637.3062	[M-H]-	C30H46N4O11	?
81	12.23	n.i.			
81	12.35	687.339	[M-H]-	C37H52O12	?
81	12.75	653.426	[M-H]-	C36H62O10	?
87	13.25	282.277	[M+H]+	C18H35NO	Fatty amide
93	14.95	255.23	[M-H]-	C16H32O2	Palmitic acid
99	15.85	n.i.			

n.d.; not ditected

n.i.; not identified parent ion

?; not annotated from database

### Effects of tomato extract, 9-oxo-ODA and daphnetin on pro-inflammatory cytokine production in LPS-stimulated macrophages

**Fig 4:**
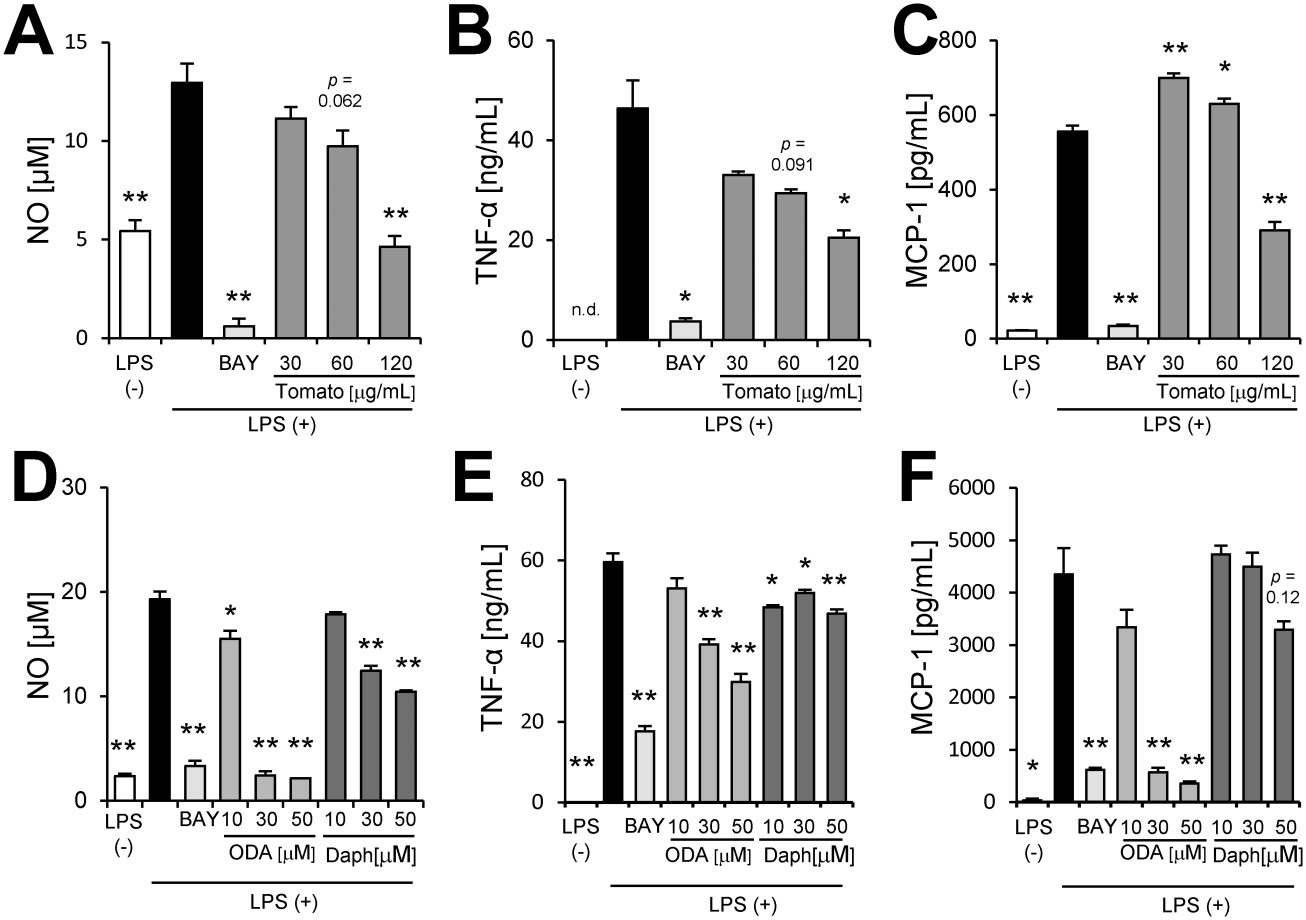
Effects of tomato extract, 9-oxo-ODA, and daphnetin on secretion of inflammatory mediators by LPS-stimulated RAW264.7 macrophages. RAW264.7 cells were stimulated with LPS (100 ng/mL) and incubated with tomato extract, 9-oxo-ODA, or daphnetin for 24 h. The levels of NO, TNF-α, and MCP-1 were measured. Effect of tomato extract on **(A)** NO, **(B)** TNF-α, and **(C)** MCP-1 secretion. Effect of 9-oxo-ODA and daphnetin on **(D)** NO, **(E)** TNF-α, and **(F)** MCP-1 secretion. Data are presented as means ± SEM (n = 3/group). *p < 0.05, **p < 0.01 vs. culture treated with LPS alone. BAY; positive control for anti-inflammatory effect.

We demonstrated that tomato extract had the ability to decrease not only NO production (Fig 4A) but also TNF-α and MCP-1 production (Fig 4B and 4C) in a dose-dependent manner. We investigated whether 9-oxo-ODA and daphnetin derived from tomato extract inhibited NO, MCP-1, and TNF-α production in activated macrophages stimulated with LPS. Our data showed that 9-oxo-ODA and daphnetin suppressed LPS-induced NO production in a dose-dependent manner (Fig 4D). In addition, we observed that the mRNA expression of *Nos2* was decreased by 9-oxo-ODA treatment (S1 Fig). Furthermore, 9-oxo-ODA inhibited LPS-induced TNF-α and MCP-1 production in a dose dependent manner (Fig 4E and 4F). Although daphnetin inhibited LPS-induced TNF-α and MCP-1 production, its effect was weaker than that of 9-oxo-ODA (Fig 4E and 4F). These results indicated that tomato extract, 9-oxo-ODA and daphnetin suppressed pro-inflammatory mediators in LPS-stimulated macrophages.

### Effects of tomato extract, 9-oxo-ODA and daphnetin on inflammation by co-culture of adipocytes and macrophages

**Fig 5:**
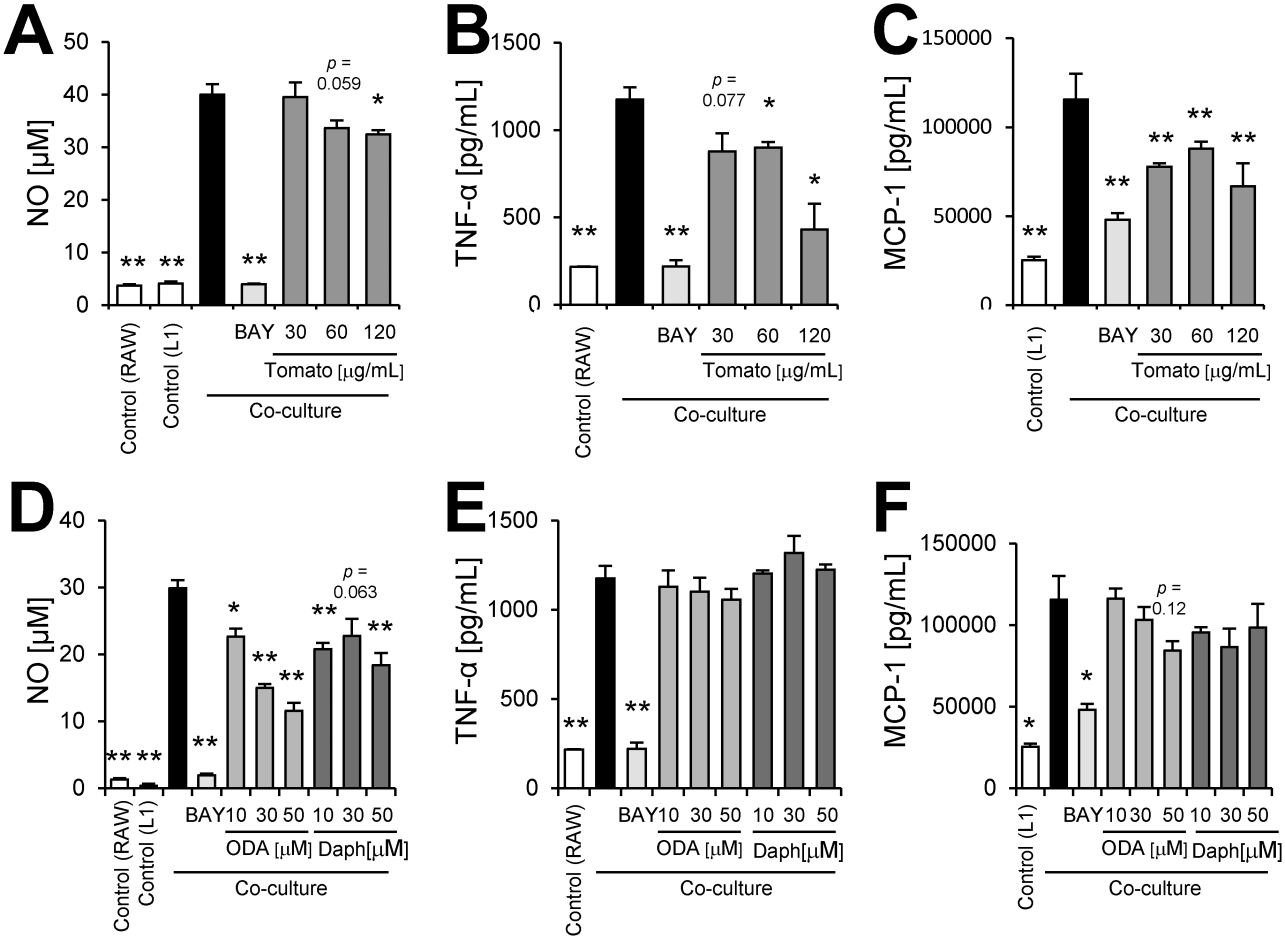
Effects of tomato extract, 9-oxo-ODA, and daphnetin on inflammation induced by co-culture of 3T3-L1 adipocytes and RAW264.7 macrophages. Differentiated 3T3-L1 adipocytes were co-cultured with RAW264.7 macrophages for 24 h. The levels of NO, TNF-α, and MCP-1 in the co-culture medium were measured. Effect of tomato extract on **(A)** NO, **(B)** TNF-α, and **(C)** MCP-1 secretion. Effect of 9-oxo-ODA and daphnetin on **(D)** NO, **(E)** TNF-α, and **(F)** MCP-1 secretion. Data are presented as means ± SEM (n = 3/group). *p < 0.05, **p < 0.01 vs. non-treated co-culture. TNF-α in control (L1) and MCP-1 in control (RAW) are low limited of quantification. BAY; positive control for anti-inflammatory effect.

The vicious cycle that augments inflammation in obese adipose tissue was mimicked by the co-culture of differentiated 3T3-L1 and RAW264.7 cells using a contact system. Indeed, the co-culture of these cells exhibited a significant increase in NO, TNF-α, and MCP-1 production (Fig 5A–5F). We demonstrated that tomato extract decreased NO, TNF-α, and MCP-1 production (Fig 5A–5C). 9-oxo-ODA and daphnetin treatment in the co-culture notably inhibited NO production (Fig 5D). Although 9-oxo-ODA and daphnetin also suppressed NO production, these compounds had no effect on TNF-α and MCP-1 production (Fig 5E and 5F). These data indicated that 9-oxo-ODA and daphnetin derived from tomato extract mainly suppressed NO production on inflammation by co-culture of adipocytes and macrophages.

### Mechanism of inhibition of pro-inflammatory mediators by tomato extract, 9-oxo-ODA and daphnetin

**Fig 6:**
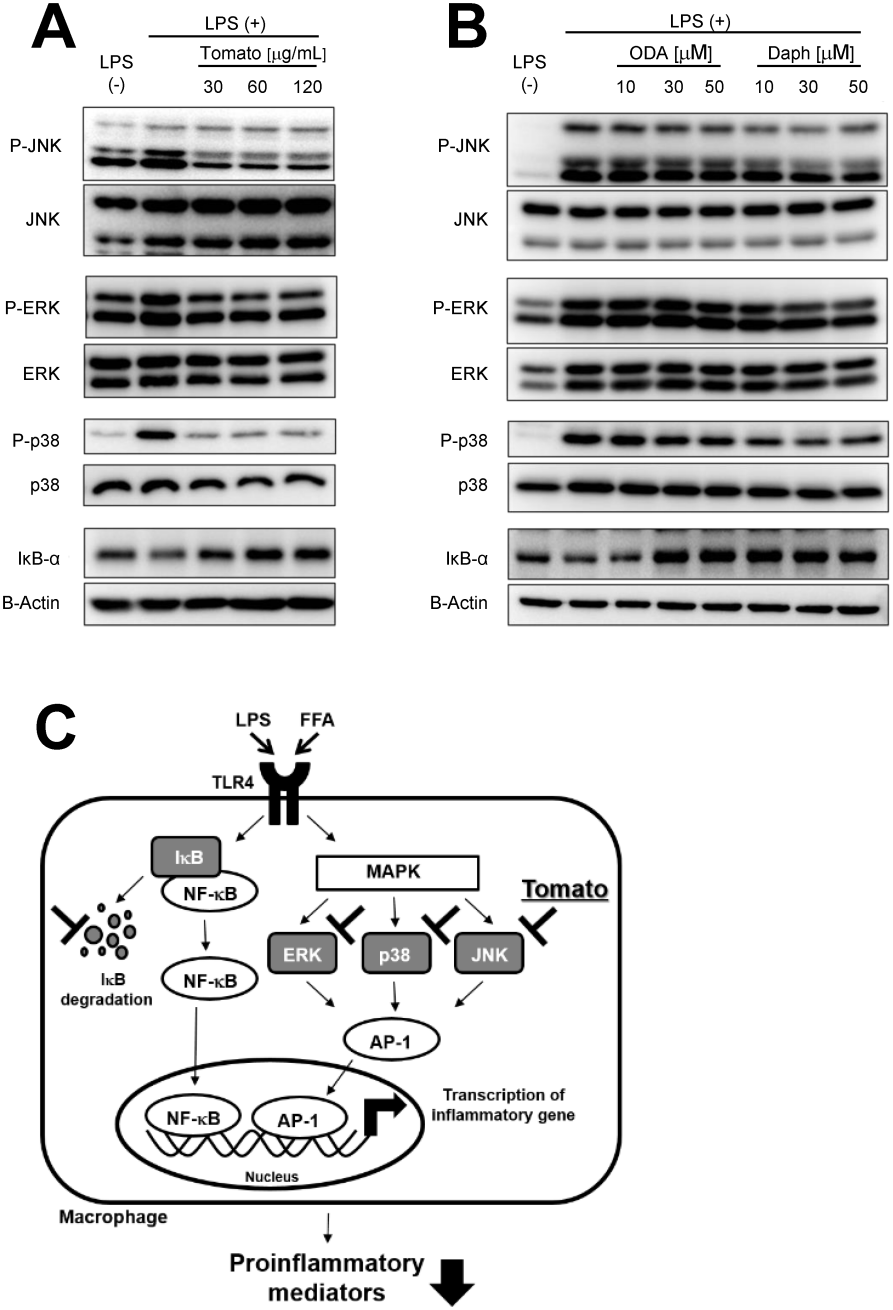
Effects of tomato extract, 9-oxo-ODA, and daphnetin on JNK, ERK, and p38 phosphorylation, and IκB-α degradation in RAW264.7 macrophages stimulated with LPS. RAW264.7 cells were stimulated with LPS (100 ng/mL) and incubated with tomato extract, 9-oxo-ODA, or daphnetin for 1 h. Total cell lysates were extracted from cultured RAW264.7 cells. Effect of **(A)** tomato extract, **(B)** 9-oxo-ODA, and daphnetin on phosphorylated JNK, ERK, and p38, and IκB-α degradation. **(C)** Schematic illustration of the mechanism of inhibition of pro-inflammatory mediators by tomato extract, 9-oxo-ODA, and daphnetin.

To clarify the mechanism of inhibition of pro-inflammatory cytokines by tomato extract, 9-oxo-ODA, and daphnetin, MAPKs (JNK, ERK, and p38) phosphorylation was examined in LPS-stimulated RAW264.7 macrophages. The LPS treatment significantly facilitated the phosphorylation of MAPKs, whereas tomato extract inhibited this phosphorylation (Fig 6A). In addition, LPS-induced IκB-α degradation, which leads to nuclear factor kappa B (NF-κB) activation, was suppressed by tomato extract (Fig 6A). The quantification of western blot signals also showed that tomato extract inhibited the phosphorylation of MAPKs and IκB-α degradation in LPS-stimulated RAW264.7 macrophages (S2A–S2F Fig). 9-oxo-ODA inhibited JNK and p38 phosphorylation, and IκB-α degradation (Fig 6B), whereas daphnetin inhibited JNK, ERK, and p38 phosphorylation, and IκB-α degradation (Fig 6B). These findings indicated that the anti-inflammatory effects of tomato extract, 9-oxo-ODA and daphnetin were through suppression of MAPKs phosphorylation and IκB-α degradation.

## Discussion

In this study, we demonstrated for the first time that tomato extract possessed many types of anti-inflammatory compounds (Fig 2F). We also showed that this extract was able to reduce plasma glucose and TG level in HFD-fed mice (Fig 1B and 1C). Previous studies have indicated that ingestion of tomatoes was related to suppression of various chronic diseases, including type 2 diabetes, cancer, and cardiovascular diseases[14–16]. These interesting effects of tomato are generally attributed to carotenoids, including lycopene[28,29]. It is well known that lycopene has anti-oxidative[30] and anti-inflammatory properties[31]. On the other hand, we showed that a large number of compounds in tomato, except carotenoids, have the ability to inhibit inflammation (Figs 2 and 3). LC-MS and database analysis showed that almost all the compounds were specified as fatty acid and coumarin derivatives from HPLC fractions of the 90% MeOH extract (Tables 5–7 and Fig 2F). In addition, we demonstrated that 9-oxo-ODA and daphnetin were identified as anti-inflammatory compounds from tomato extract (Fig 3). Furthermore, 9-oxo-ODA, daphnetin and lycopene had an ability to reduce inflammation on NO secretion (S3 Fig). Our results and previous findings raise the possibility that the effect of tomato on inflammation is explained by factors other than carotenoids.

9-oxo-ODA is an oxylipin and an linoleic acid (LIA) derivative. There is a possibility that enzymatic reaction participates in the production of 9-oxo-ODA[32]. It has been reported that free fatty acids are the substrates for lipoxygenases (LOXs)[33] and that 9-LOX activity oxidizes LIA at the C9 position to produce 9-hydroperoxy octadecatrienoic acid[34,35], which are possible precursors of 9-oxo-ODA. In a previous study, we have reported that 9-oxo-ODA activated PPARα[19]. It has been reported that PPARα promoted β-oxidation via enhancement of its target gene expression[36–38]. This resulted in reduced fat storage[39,40]. Therefore, PPARα is important for the regulation of lipid metabolism. In this study, we showed that plasma TG level decreased by tomato extract treatment. Probably, 9-oxo-ODA was involved in this process. On the other hand, we demonstrated for the first time that 9-oxo-ODA was not only a PPARα activator but also showed anti-inflammatory properties. In addition, previous studies have reported that PPARγ the subtype of PPARα, is likely to be concerned with anti-inflammatory effect in LPS-stimulated macrophages[41,42]. Therefore, we investigated whether the anti-inflammatory activities of 9-oxo-ODA participated in PPARα and PPARγ by using antagonist with reference to the previous studies[19,42]. The anti-inflammatory activities of 9-oxo-ODA on NO production was unchanged by PPARα antagonist GW6471 and PPARγ antagonist GW9662 (S4A and S4B Fig). Although our findings raise the possibility that 9-oxo-ODA is not related to PPARα and PPARγ on the inhibition of NO secretion, further examination is necessary to elucidate the anti-inflammatory activities of 9-oxo-ODA via PPARα and PPARγ. Our previous and present studies showed the functional diversity of 9-oxo-ODA and suggested that 9-oxo-ODA contributed to the effect of tomato on health maintenance. In the tomato extract and white adipose tissue sample, we analyzed 9-oxo-ODA, which had the strongest effect on NO secretion in LPS-stimulated RAW264.7 macrophages (Fig 3H) using LC-MS. 9-oxo-ODA was detected in the tomato extract (Rt = 8.93 min, S1A Fig). The amount of 9-oxo-ODA in the tomato extract was approximately 160 ng/mg (data not shown). Furthermore, 9-oxo-ODA was also detected in the white adipose tissue and tended to increase in presence of the tomato extract treatment (HFD group: approximately 35 ng/mg WAT; Tomato extract group: approximately 60 ng/mg WAT; S1B Fig). We estimated that 9-oxo-ODA present at low level in HFD mice was an endogenous metabolite. The detection of 9-oxo-ODA in the white adipose tissue suggested that this compound acted directly as an anti-inflammatory factor.

In this study, we demonstrated that daphnetin and esculetin were present in tomato extract (Fig 3). Daphnetin, a natural coumarin derivative, is isolated from the traditional Chinese medicinal herb *Daphne odora var*. *marginata* (*D*. *marginata*)[43]. In this work, although we detected many types of coumarin (Table 6 and Fig 3), only two compounds (daphnetin and esculetin) were able to inhibit inflammation (Fig 3H). It has been reported that these compounds inhibit inflammation[44,45]. These compounds have two neighboring OH-groups (Fig 3I). The neighboring OH-groups may be important for the anti-inflammatory effect. On the other hand, 4-methoxycoumarin, 4-hydroxycoumarin, and 7-hydroxy-4-methylcoumarin, which were identified from NO inhibitory fractions, had no effect on the suppression of NO production (Fig 3H). ESI, a common method for metabolite analysis using LC-MS, was used for the identification of active compounds. We surmised that these fractions were likely to contain other anti-inflammatory compounds, which were difficult to detect under ESI condition.

Previous studies have reported that chronic inflammation in the adipose tissue was important for obesity[46] and that NF-κB[47] and MAPKs[48] were key factors of inflammation. NF-κB is present in the cytoplasm in an inactive form owing to the binding to IκB-α[49]. IκB-α degradation induces NF-κB translocation to the nucleus, resulting in the secretion of pro-inflammatory factors[49]. Therefore, IκB-α degradation is involved in regulating obesity-related inflammation[50,51]. MAPKs include three major groups: ERK, JNK, and p38 kinase[48]. MAPKs induce activation of AP-1 transcription factor, which stimulates the expression of inflammatory cytokine genes alone or in combination with NF-κB[48]. These findings suggest that NF-κB and MAPKs are important for activation of obesity-related inflammation. In this study, we demonstrated that tomato extract, 9-oxo-ODA and daphnetin had the ability to inhibit IκB-α degradation and phosphorylation of MAPKs (Fig 6B and 6C).

In Fig 5, although tomato extract inhibited NO, TNF-α, and MCP-1 secretion, 9-oxo-ODA and daphnetin had no effect on TNF-α and MCP-1 secretion. These findings suggested that other compounds in tomato were likely to contribute to decrease TNF-α and MCP-1 secretion. On the other hand, tomato extract, 9-oxo-ODA, and daphnetin inhibited NO secretion in the co-culture (Fig 5). In a previous study, it has been reported that the suppression of NO secretion contributes to improve glucose metabolism disorder[52]. We demonstrated that the tomato extract decreased plasma glucose level and the expression of *Nos2* involved in NO production (Fig 1). Both our results and previous findings raise the possibility that tomato extract, including 9-oxo-ODA and daphnetin, partly decreases plasma glucose level via suppression of NO secretion.

Whereas we identified 9-oxo-ODA and daphnetin as anti-inflammatory compounds from tomato extract, we also suggested that many other anti-inflammatory compounds remain to be identified. Not only the effect of a few compounds alone but also the additive or synergistic effect of many compounds should be taken into consideration when discussing more appropriate food function. In fact, many metabolites are contained in tomato fruit[53]. Therefore, in this study we attempted to estimate many anti-inflammatory compounds by wide-range screening using LC-MS. In consequence, we showed that a large number of anti-inflammatory compounds may exist in tomato. Moreover, specified as fatty acid and coumarin derivatives were identified as anti-inflammatory compounds. Even though this study provides novel insights into the estimation of food function, further studies are necessary to elucidate the relationship between these compounds and the effect of tomato on inflammation. In conclusion, wide-range screening using LC-MS and NO assay revealed that tomato possessed many anti-inflammatory compounds. In particular, 9-oxo-ODA and daphnetin inhibited the secretion of inflammatory cytokines via the suppression of NF-κB and MAPKs pathway. Furthermore, our study suggested that the effect of tomato on suppression of plasma glucose and expression of *Nos2* in the white adipose tissue was partly caused by 9-oxo-ODA.

## Supporting information

S1 Fig Effect of 9-oxo-ODA and daphnetin on *Nos2* mRNA expression levels by LPS-stimulated RAW264.7 macrophage. RAW264.7 cells were stimulated with LPS (100 ng/mL) and incubated with 9-oxo-ODA or daphnetin (50 μM) for 24 h. The levels of *Nos*2 mRNA expression were measured. Data are presented as means ± SEM (n = 4–6/group). *p < 0.05 vs. culture treated with LPS alone.
(PPTX)

S2 Fig The quantification of western blot signals on MAPKs phosphorylation and IκB-α degradation in LPS-stimulated RAW264.7 macrophages treated with tomato extract. RAW264.7 cells were stimulated with LPS (100 ng/mL) and incubated with tomato extract for 1h. Total cell lysates were extracted from cultured RAW264.7 cells. The quantification of western blot signals on **(A)** JNK1, **(B)** JNK2/3, **(C)** ERK1, **(D)** ERK2, **(E)** p38 phosphorylation, and **(F)** IκB-α degradation. Data are presented as means ± SEM (n = 3–4/group). *p < 0.05, **p < 0.01 vs. culture treated with LPS alone.
(PPTX)

S3 Fig Effect of 9-oxo-ODA, daphnetin and lycopene on secretion of NO by LPS-stimulated RAW264.7 macrophage. RAW264.7 cells were stimulated with LPS (100 ng/mL) and incubated with 9-oxo-ODA, daphnetin and lycopene (30 μM) for 24 h. The levels of NO secretion were measured. Data are presented as means ± SEM (n = 3/group). *p < 0.05, **p < 0.01 vs. culture treated with LPS alone.
(PPTX)

S4 Fig Effect of 9-oxo-ODA on secretion of NO by LPS-stimulated RAW264.7 macrophage treated with or without PPARα, PPARγ antagonist. LPS-stimulated (100 ng/mL) RAW264.7 cells were incubated with 9-oxo-ODA (30 μM) and treated with or without **(A)** GW6471(10 μM), **(B)** GW9662 (10 μM) for 24h. GW6471 is a PPARα antagonist. GW9662 is a PPARγ antagonist. Data are presented as means ± SEM (n = 3/group). n.s.; Not significant vs. culture treated with LPS and 9-oxo-ODA.
(PPTX)

S5 Fig LC-MS analysis of 9-oxo-ODA in tomato extract and white adipose tissue. **(A)** The extracted ion chromatogram (m/z = 293.209) in tomato extract sample. **(B)** The amount of 9-oxo-ODA in white adipose tissue. Data are presented as means ± SEM (n = 8–10/group).
(PPTX)

## References

[1] Reilly M P, Rader D J, The Metabolic Syndrome: more than the sum of its parts? *Circulation*, 2003, 108 (13), 1546–1551. doi: 10.1161/01.CIR.0000088846.10655.E0 14517150 10.1161/01.CIR.0000088846.10655.E0

[2] Miranda P J, DeFronzo R A, Califf R M, Guyto J R, Metabolic syndrome: definition, pathophysiology, and mechanism. *American Heart J*., 2005, 149 (1), 33–45. 10.1016/j.ahj.2004.07.013 15660032

[3] Gregor M F, Hotamisligil G S, Inflammatory mechanisms in obesity. *Annu*. *Rev*. *Immunol*, 2011, 29, 415–445. doi: 10.1146/annurev-immunol-031210-101322 21219177 10.1146/annurev-immunol-031210-101322

[4] Heibornn L K, Chambell L V, Adipose tissue macrophages, low grade inflammation and insulin resistance in human obesity. *Current Pharmaceutical Design*, 2008, 14 (12), 1225–1230. 18473870 10.2174/138161208784246153

[5] Schenk S, Saberi M, Olefsky J M, Insulin sensitivity: modulation by nutrients and inflammation. *Journal of Clinical Investigation*, 2008, 118 (9), 2992–3002. doi: 10.1172/JCI34260 18769626 10.1172/JCI34260 PMC2522344

[6] Shoelson S E, Lee J, Goldfine A B, Inflammation and insulin resistance. *J*. *Clin*. *Invest*. 2006, 116 (8), 1793–1801. 16823477 10.1172/JCI29069 PMC1483173

[7] Weisberg S P, McCann D, Desai M, Rosenbaum M, Leibel R L, Ferrante A W, Obesity is associated with mavrophage accumulation in adipose tissue. *J*. *Clin*. *Invest*. 2003, 112 (12), 1796–1808. doi: 10.1172/JCI19246 14679176 10.1172/JCI19246 PMC296995

[8] Xu H, Barnes G T, Yang Q, Tan G, Yang D, Chou C J, et al Chronic inflammation in fat plays a crucial role in the development of obesity-related insulin resistance. *J*. *Clin*. *Invest*. 2003, 112 (12), 1821–1830. doi: 10.1172/JCI19451 14679177 10.1172/JCI19451 PMC296998

[9] Kanda H, Tateya S, Tamori Y, Kotani K, Hiasa K, Kitazawa R, et al MCP-1 contributes to macrophage infiltration into adipose tissue, insulin resistance, and hepatic steatosis in obesity *J*. *Clin*. *Invest*. 2006, 116 (6), 1494–1505. doi: 10.1172/JCI26498 16691291 10.1172/JCI26498 PMC1459069

[10] Hotamisligil G S, Shargill N S, Spiegelman B M, Adipose expression of tumor necrosis factor-α: direct role in obesity-linked insulin resistance. *Science*, 1993, 259 (5091), 87–91. 7678183 10.1126/science.7678183

[11] Engeli S, Boschmann M, Adams F, Franke G, Gorzelniak K, Janke J, et al Dissociation between adipose nitric oxide synthase expression and tissue metabolism. *J*. *Clin*. *Endocrinol*. *Metab*. 2007, 92 (7), 2706–2711. doi: 10.1210/jc.2007-0234 17456572 10.1210/jc.2007-0234

[12] Suganami T, Nishida J, Ogawa Y, A paracrine loop between adipocytes and macrophages aggravates inflammatory changes: role of free fatty acids and tumor necrosis factor-α *Arterioscler Thromb*. *Vasc*. *Biol*. 2005, 25 (10), 2062–2068. doi: 10.1161/01.ATV.0000183883.72263.13 16123319 10.1161/01.ATV.0000183883.72263.13

[13] Odegaard J I, Chawla A, Pleiotropic actions of insulin resistance and inflammation in metabolic homeostasis *Science*, 2013, 339 (6116), 172–177. doi: 10.1126/science.1230721 23307735 10.1126/science.1230721 PMC3725457

[14] Lazarus S A, Bowen K, Garg M L, Tomato juice and platelet aggregation in type 2 diabetes. *JAMA* 2004, 292 (7), 805–806. doi: 10.1001/jama.292.7.805 15315994 10.1001/jama.292.7.805

[15] Blum A, Monir M, Wirsansky I, Ben-Arzi S, The benefical effects of tomatoes. *European Journal of Internal Medicine*, 2005, 16 (6), 402–404. doi: 10.1016/j.ejim.2005.02.017 16198897 10.1016/j.ejim.2005.02.017

[16] Kirstie C A, Jessica K C, Susan Z, Elizabeth H J, John W E, The Tomato As a Functional Food. *American Society for Nutritional Sciences*, 2005, 135 (5), 1226–1230.

[17] Ghavipour M, Saedisomeolia A, Dialali M, Sotoudeh G, Eshraghyan M R, Moghadam A M, et al Tomato juice consumption reduces systemic inflammation in overweight and obese females. *Br*. *J*. *Nutr*. 2013, 109 (11), 2031–2035. doi: 10.1017/S0007114512004278 23069270 10.1017/S0007114512004278

[18] Li Y F, Chang Y Y, Huang H C, Wu Y C, Yang M D, Chao P M, Tomato juice supplementation in young women reduces inflammatory adipokine levels independently of body fat reduction. *Nutrition*, 2015, 31 (5), 691–696. doi: 10.1016/j.nut.2014.11.008 25837214 10.1016/j.nut.2014.11.008

[19] Kim Y I, Hirai S, Takahashi H, Goto T, Ohyane C, Tsugane T, et al 9-oxo-10(E),12(E)-Octadecadienoic acid derived from tomato is a potent PPARα agonist to decrease triglyceride accumulation in mouse primary hepatocytes. *Mol*. *Nutr*. *Food Res*. 2011, 55 (4), 585–593. doi: 10.1002/mnfr.201000264 21462326 10.1002/mnfr.201000264

[20] Kim Y I, Hirai S, Goto T, Ohyane C, Takahashi H, Tsugane T, et al Potent PPARα activator derived from tomato juice, 13-oxo-9,11-octadecadienoic acid, decreases plasma and hepatic triglyceride in obese diabetic mice. *PLoS One* 2012, 7 (2), e31317 doi: 10.1371/journal.pone.0031317 22347463 10.1371/journal.pone.0031317 PMC3276502

[21] Takahashi H, Kamakari K, Goto T, Hara H, Mohri S, Suzuki H, et al 9-oxo-10(E),12(Z),15(Z)- octadecatrienoic acid activates peroxisome proliferator-activated receptor-α in hepatocytes. *Lipids*. 2015, 50 (11), 1083–1091. doi: 10.1007/s11745-015-4071-3 26387026 10.1007/s11745-015-4071-3

[22] Escher P, Wahli W, Peroxisome proliferator-activated receptors: insight into multiple cellular functions. *Mutat*. *Res*. 2000, 448 (2), 121–138. 10725467 10.1016/s0027-5107(99)00231-6

[23] Desvergne B, Wahli W, Peroxisome proliferator-activated receptors: nuclear control of metabolism. *Endocr*. *Rev*. 1999, 20 (5), 649–688. doi: 10.1210/edrv.20.5.0380 10529898 10.1210/edrv.20.5.0380

[24] Chinetti G, Fruchart J C, Staeles B, Peroxisome proliferator-activated receptors (PPARs): nuclear receptors at the crossroads between lipid metabolism and inflammation. *Inflamm*. *Res*. 2000, 49 (10), 497–505. doi: 10.1007/s000110050622 11089900 10.1007/s000110050622

[25] Takahashi H, Goto T, Yamazaki Y, Kamakari K, Hirata M, Suzuki H, et al Metabolomics reveal 1-palmitoyl lysophosphatidylcholine production by peroxisome proliferator-activated receptor α. *J*. *Lipid Res*. 2015, 56 (2), 254–265. doi: 10.1194/jlr.M052464 25510248 10.1194/jlr.M052464 PMC4306680

[26] Li Y, Goto T, Ikutani R, Lin S, Takahashi N, Takahashi H, et al Xanthoangelol and 4-hydroxyderrcin suppress obesity-induced inflammatory responses. *Obesity (Silver Spring)*. 2016, 24 (11), 2351–2360. 27619735 10.1002/oby.21611

[27] Granger D L, Taintor R R, Boockvar K S, Hibbs J B, Measurement of nitrate and nitrite in biological samples using nitrate reductase and Griess reaction. *Methods Enzymol*. 1996, 268, 142–151. 8782580 10.1016/s0076-6879(96)68016-1

[28] Gouranton E, Thabuis C, Riollet C, Malezet-Desmoulins C, E l Yazidi C, Amiot M J, et al Lycopene inhibits proinflammatory cytokine and chemokine expression in adipose tissue. *J*. *Nutr*. *Biochem*. 2011, 22 (7), 642–648. doi: 10.1016/j.jnutbio.2010.04.016 20952175 10.1016/j.jnutbio.2010.04.016

[29] Luvizotto Rde A, Nascimento A F, Imaizumi E, Pierine D T, Conde S J, Correa C R, et al Lycopene supplementation modulates plasma concentrations and epididymal adipose tissue mRNA of leptin, resistin and IL-6 in diet-induced obese rats. *Br*. *J*. *Nutr*. 2013, 110 (10), 1803–1809. doi: 10.1017/S0007114513001256 23632237 10.1017/S0007114513001256

[30] Palozza P, Parrone N, Catalano A, Simone R, Tomato lycopene and inflammatory cascade: basic interactions and clinical implications. *Curr*. *Med*. *Chem*. 2010, 17 (23), 2547–2563. 20491642 10.2174/092986710791556041

[31] Fenni S, Hammou H, Astier J, Bonnet L, Karkeni E, Couturier C, et al Lycopene and tomato powder supplementation similarly inhibit high-fat diet induced obesity, inflammatory response, and associated metabolic disorders. *Mol*. *Nutr*. *Food Res*. 2017, Epub ahead of print. 10.1002/mnfr.201601083 28267248

[32] Takahashi H, Kamakari K, Suzuki H, Mohri S, Goto T, Takahashi N, et al Localization of 9- and 13-oxo-octadecadienoic acids in tomato fruit. *Biosci*. *Biotechnol*. *Biochem*. 2014, 78 (10), 1761–1764. doi: 10.1080/09168451.2014.930330 25060034 10.1080/09168451.2014.930330

[33] Pulvera Z, M, Kitamura K, Hajika M, Shimada K, Matsui K, Oxylipin metabolism in soybean seeds containing different sets of lipoxygenase isozymes after homogenization. *Biosci*. *Biotechnol*. *Biochem*. 2006, 70 (11), 2598–2603. 17090947 10.1271/bbb.60121

[34] Kuo J M, Hwang A, Yeh D B, Pan M H, Tsai M L, Pan B S, Lipoxygenase from banana leaf: purification and characterization of an enzyme that catalyzes linoleic acid oxygenation at the 9-position. *J*. *Agric*. *Food Chem*. 2006, 54 (8), 3151–3156. doi: 10.1021/jf060022q 16608245 10.1021/jf060022q

[35] Mariutto M, Fauconnier M L, Ongena M, Laloux M, Wathelet J P, du Jardin P, et al Reprogramming of fatty acid and oxylipin synthesis in rhizobacteria-induced systemic resistance in tomato. *Plant Mol*. *Biol*. 2014, 84 (4–5), 455–467. doi: 10.1007/s11103-013-0144-y 24146221 10.1007/s11103-013-0144-y

[36] Tugwood J D, Issemann I, Anderson R G, Bundell K R, McPheat W L, Green S, The mouse peroxisome proliferator activated receptor recognizes a response element in the 5' flanking sequence of the rat acyl CoA oxidase gene. *EMBO J*. 1992, 11 (2), 433–439. 1537328 10.1002/j.1460-2075.1992.tb05072.x PMC556472

[37] Roepstorff C, Halberg N, Hillig T, Saha A K, Ruderman N B, Wojtaszewski J F, et al Malonyl-CoA and carnitine in regulation of fat oxidation in human skeletal muscle during exercise. *Am*. *J*. *Physiol*. *Endocrinol*. *Metab*. 2005, 288 (1), E133–142. doi: 10.1152/ajpendo.00379.2004 15383373 10.1152/ajpendo.00379.2004

[38] Miller C W, Ntambi J M, Peroxisome proliferators induce mouse liver stearoyl-CoA desaturase 1 gene expression. *Proc*. *Natl*. *Acad*. *Sci*. *USA*. 1996, 93 (18), 9443–9448. 8790349 10.1073/pnas.93.18.9443 PMC38447

[39] Bogna G G, Peroxisome proliferator-activated receptors and their ligands: nutritional and clinical implications. *Nutr*. *J*. 2014, 13, 17 doi: 10.1186/1475-2891-13-17 24524207 10.1186/1475-2891-13-17 PMC3943808

[40] Badman M K, Pissios P, Kennedy A R, Koukos G, Flier J S, Maratos-Flier E, Hepatic fibroblast growth factor 21 is regulated by PPARalpha and is a key mediator of hepatic lipid metabolism in ketotic states. *Cell Metab*. 2007, 5 (6), 426–437. doi: 10.1016/j.cmet.2007.05.002 17550778 10.1016/j.cmet.2007.05.002

[41] Kang M S, Hirai S, Goto T, Kuroyanagi K, Lee J Y, Uemura T, et al Dehydroabietic acid, a phytochemical, acts as ligand for PPARs in macrophages and adipocytes to regulate inflammation. *Biochem Biophys Res Commun*. 2008, 369 (2), 333–338. doi: 10.1016/j.bbrc.2008.02.002 18267111 10.1016/j.bbrc.2008.02.002

[42] Lin C H, Lee S Y, Zhang C C, Du Y F, Hung H C, Wu H T, et al Fenretinide inhibits macrophage inflammatory mediators and controls hypertension in spontaneously hypertensive rats via the peroxisome proliferator-activated receptor gamma pathway. *Drug Des Devel Ther*. 2016, 10, 3591–3597. doi: 10.2147/DDDT.S114879 27843299 10.2147/DDDT.S114879 PMC5098527

[43] Yang E B, Zhao Y N, Zhang K, Mack P, Daphnetin, one of coumarin derivatives, is a protein kinase inhibitor. *Biochem Biophys Res Commun*. 1999, 260 (3), 682–685. doi: 10.1006/bbrc.1999.0958 10403826 10.1006/bbrc.1999.0958

[44] Yu W, Wang H, Ying H, Yu Y, Chen D, Ge W, et al Daphnetin attenuates microglial activation and proinflammatory factor production via multiple signaling pathways. Int Immunopharmacol. 2014, 21 (1), 1–9. doi: 10.1016/j.intimp.2014.04.005 24747094 10.1016/j.intimp.2014.04.005

[45] Kim Y, Park Y, Namkoong S, Lee J, Esculetin inhibits the inflammatory response by inducing heme oxygenase-1 in cocultured macrophages and adipocytes. *Food Funct*. 2014, 5 (9), 2371–2377. doi: 10.1039/c4fo00351a 25088305 10.1039/c4fo00351a

[46] Wensveen F M, Valentić S, Šestan M, Turk Wensveen T, Polić B, The "Big Bang" in obese fat: Events initiating obesity-induced adipose tissue inflammation. *Eur*. *J*. *Immunol*. 2015, 45 (9), 2446–2456. doi: 10.1002/eji.201545502 26220361 10.1002/eji.201545502

[47] Benzler J, Ganjam G K, Pretz D, Oelkrug R, Koch C E, Legler K, et al Central inhibition of IKKα/NF-κB signaling attenuates high-fat diet-induced obesity and glucose intolerance. *Diabetes*. 2015, 64 (6), 2015–2027. doi: 10.2337/db14-0093 25626735 10.2337/db14-0093

[48] Pearson G, Robinson F, Beers Gibson T, Xu B E, Karandikar M, Berman K, et al Mitogen-activated protein (MAP) kinase pathways: regulation and physiological functions. *Endocr*. *Rev*. 2001, 22 (2), 153–183. doi: 10.1210/edrv.22.2.0428 11294822 10.1210/edrv.22.2.0428

[49] Karin M, Yamamoto Y, Wang Q M, The IKK NF-kappa B system: a treasure trove for drug development. *Nat*. *Rev*. *Drug Discov*. 2004, 3 (1), 17–26. doi: 10.1038/nrd1279 14708018 10.1038/nrd1279

[50] Suganami T, Tanimoto-Koyama K, Nishida J, Itoh M, Yuan X, Mizuarai S, et al Role of the Toll-like receptor 4/NF-kappaB pathway in saturated fatty acid-induced inflammatory changes in the interaction between adipocytes and macrophages. *Arterioscler*. *Thromb*. *Vasc*. *Biol*. 2007, 27 (1), 84–91. doi: 10.1161/01.ATV.0000251608.09329.9a 17082484 10.1161/01.ATV.0000251608.09329.9a

[51] Ravi R, Mookerjee B, van Hensbergen Y, Bedi G C, Giordano A, El-Deiry W S, et al p53-mediated repression of nuclear factor-kappaB RelA via the transcriptional integrator p300. *Cancer Res*. 1998, 58 (20), 4531–4536. 9788595

[52] Tsuchiya K, Sakai H, Suzuki N, Iwashima F, Yoshimoto T, Shichiri M, et al Chronic blockade of nitric oxide synthesis reduces adiposity and improves insulin resistance in high fat-induced obese mice. *Endocrinology*. 2007, 148 (10), 4548–4556. doi: 10.1210/en.2006-1371 17584959 10.1210/en.2006-1371

[53] Iijima Y, Nakamura Y, Ogata Y, Tanaka K, Sakurai N, Suda K, et al Metabolite annotations based on the integration of mass spectral information. *Plant J*. 2008, 54 (5), 949–62. doi: 10.1111/j.1365-313X.2008.03434.x 18266924 10.1111/j.1365-313X.2008.03434.x PMC2440531

