# Supplementary figures and images for: Wide-range screening of anti-inflammatory compounds in tomato using LC-MS and elucidating the mechanism of their functions
Source: PLoS One. 2018 Jan 12;13(1):e0191203. doi: 10.1371/journal.pone.0191203 (PMC5766234; doi:10.1371/journal.pone.0191203)

## Slide 1
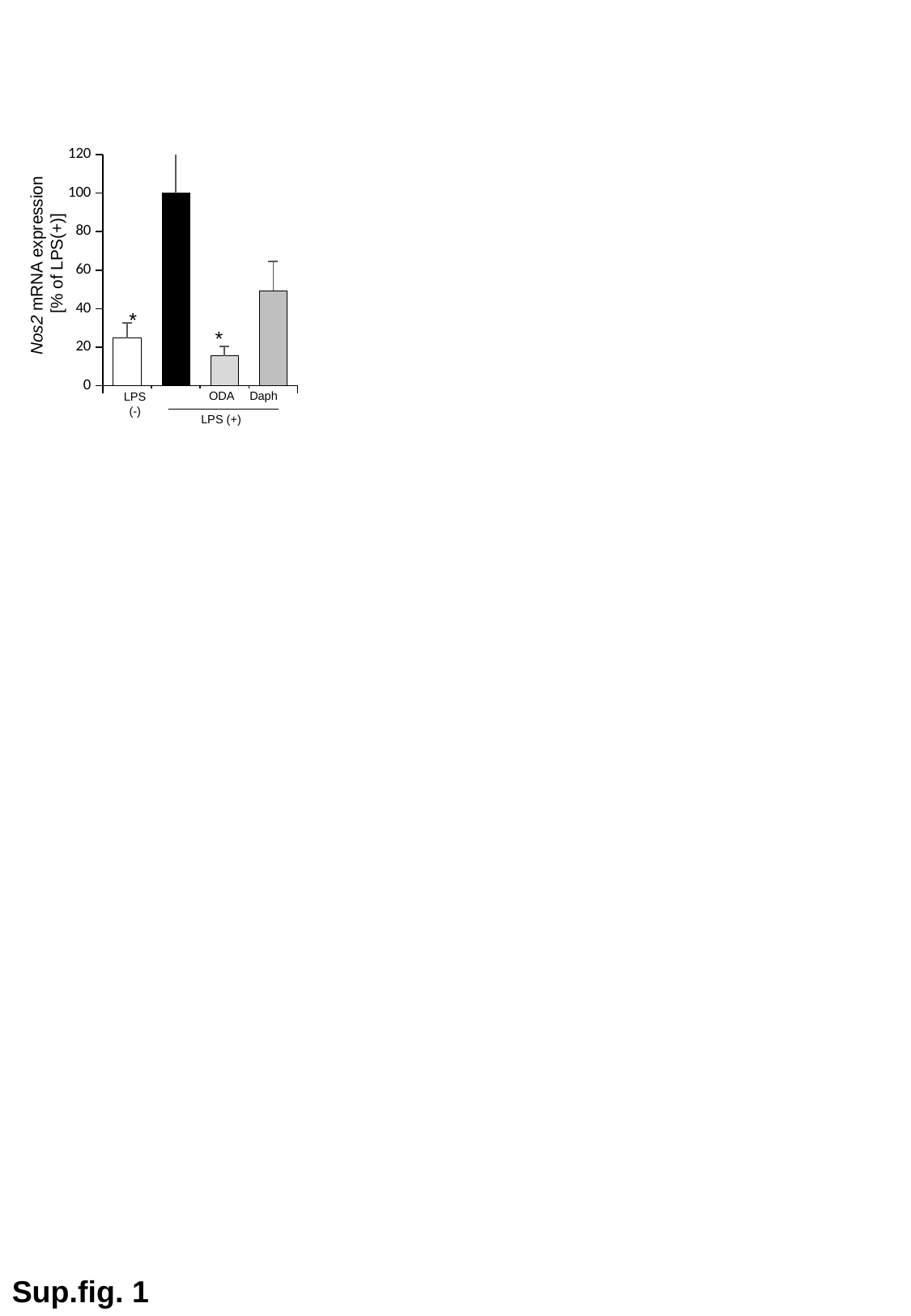

### Chart
| Category | |
|---|---|Nos2 mRNA expression
 [% of LPS(+)]
*
*
ODA
Daph
LPS
(-)
LPS (+)
Sup.fig. 1

Supplement: S1 Fig — RAW264.7 cells were stimulated with LPS (100 ng/mL) and incubated with 9-oxo-ODA or daphnetin (50 μM) for 24 h. The levels of Nos2 mRNA expression were measured. Data are presented as means ± SEM (n = 4–6/group). *p < 0.05 vs. culture treated with LPS alone. (PPTX) [file pone.0191203.s001.pptx]

## Slide 1
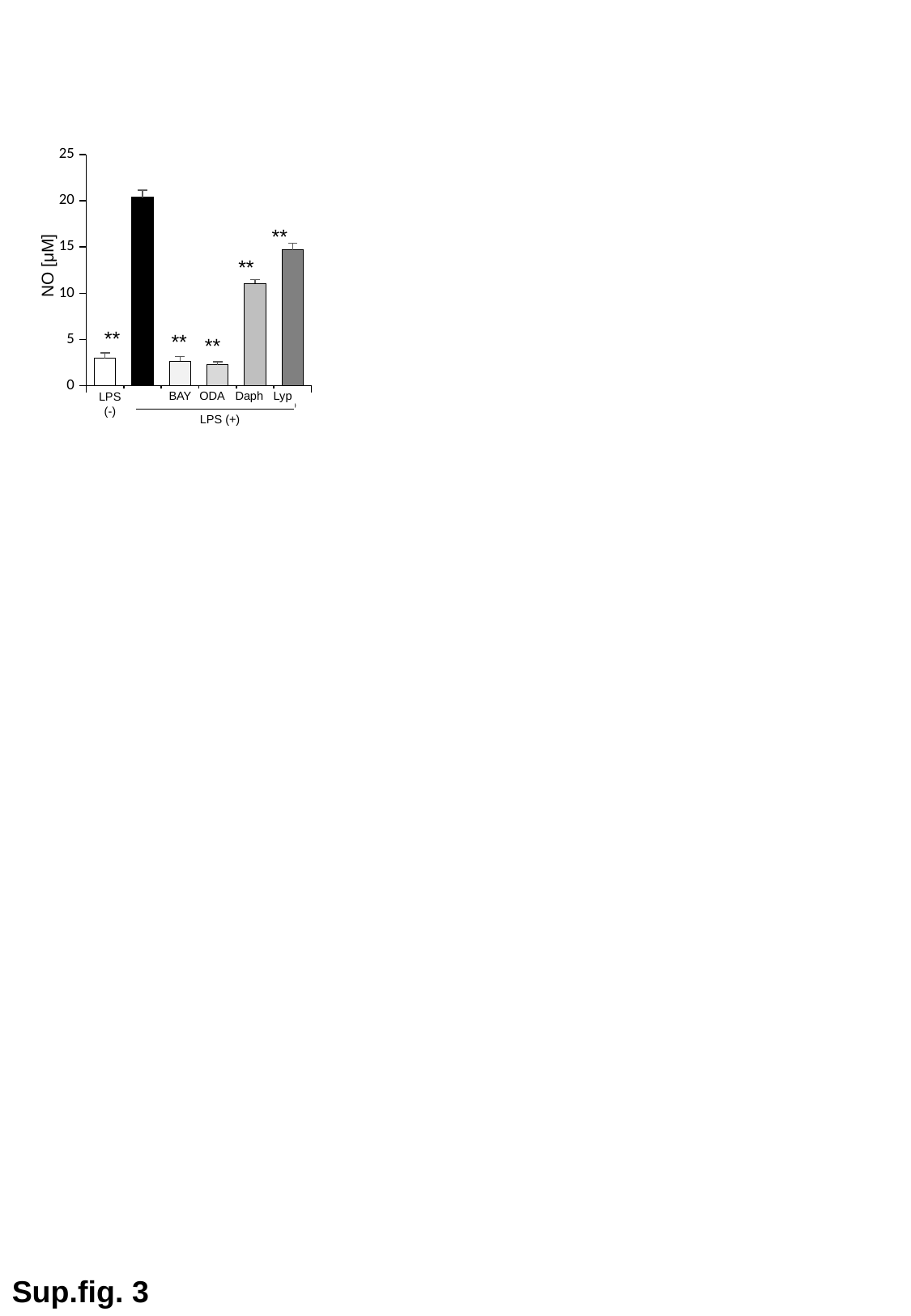

### Chart
| Category | |
|---|---|**
**
NO [μM]
**
**
**
BAY
ODA
Daph
Lyp
LPS
(-)
LPS (+)
Sup.fig. 3

Supplement: S3 Fig — RAW264.7 cells were stimulated with LPS (100 ng/mL) and incubated with 9-oxo-ODA, daphnetin and lycopene (30 μM) for 24 h. The levels of NO secretion were measured. Data are presented as means ± SEM (n = 3/group). *p < 0.05, **p < 0.01 vs. culture treated with LPS alone. (PPTX) [file pone.0191203.s003.pptx]

## Slide 1
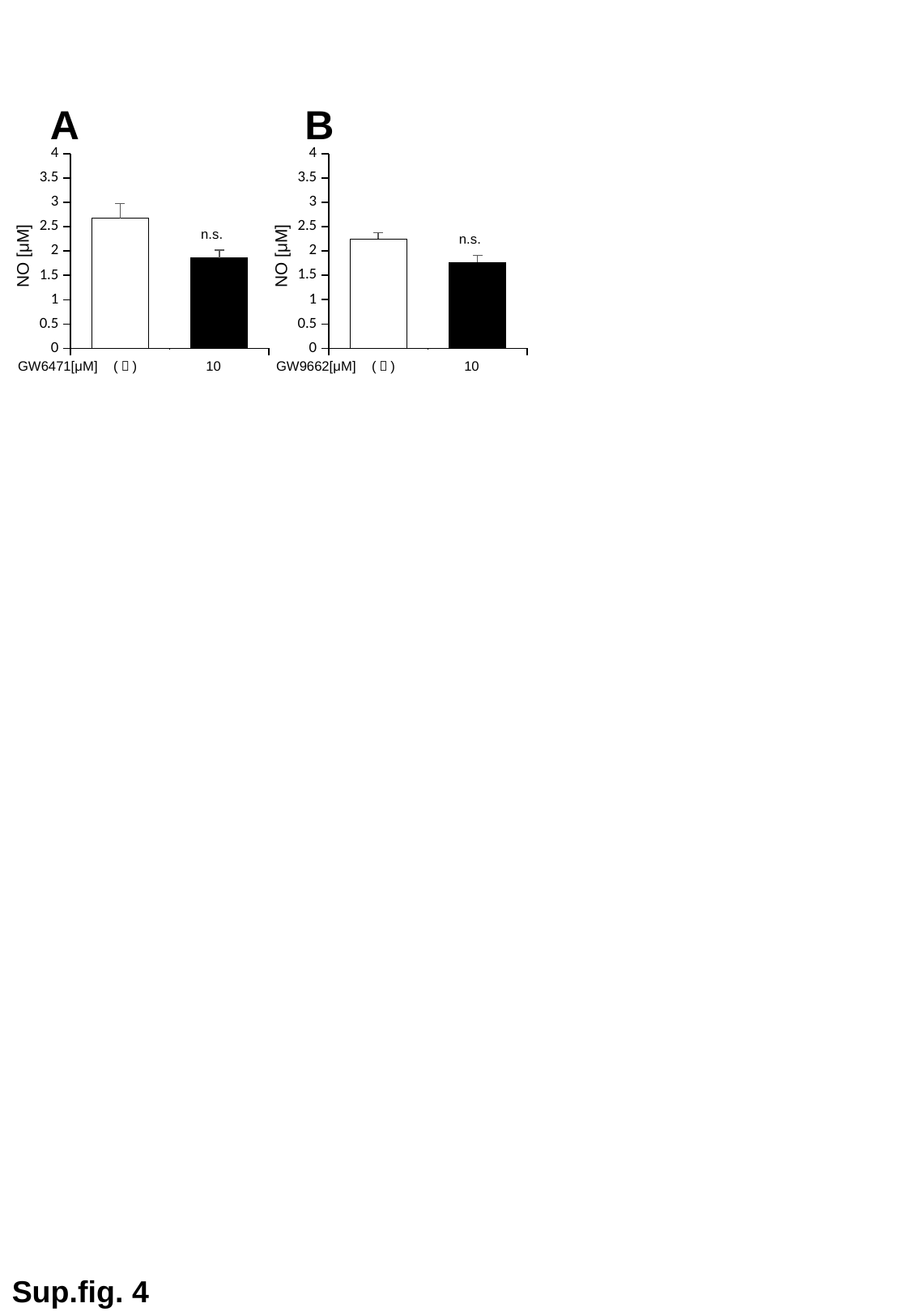

A
B
### Chart
| Category | |
|---|---|n.s.
NO [μM]
(－) 10
GW6471[μM]
### Chart
| Category | |
|---|---|n.s.
NO [μM]
GW9662[μM]
(－) 10
Sup.fig. 4

Supplement: S4 Fig — LPS-stimulated (100 ng/mL) RAW264.7 cells were incubated with 9-oxo-ODA (30 μM) and treated with or without (A) GW6471(10 μM), (B) GW9662 (10 μM) for 24h. GW6471 is a PPARα antagonist. GW9662 is a PPARγ antagonist. Data are presented as means ± SEM (n = 3/group). n.s.; Not significant vs. culture treated with LPS and 9-oxo-ODA. (PPTX) [file pone.0191203.s004.pptx]

## Slide 1
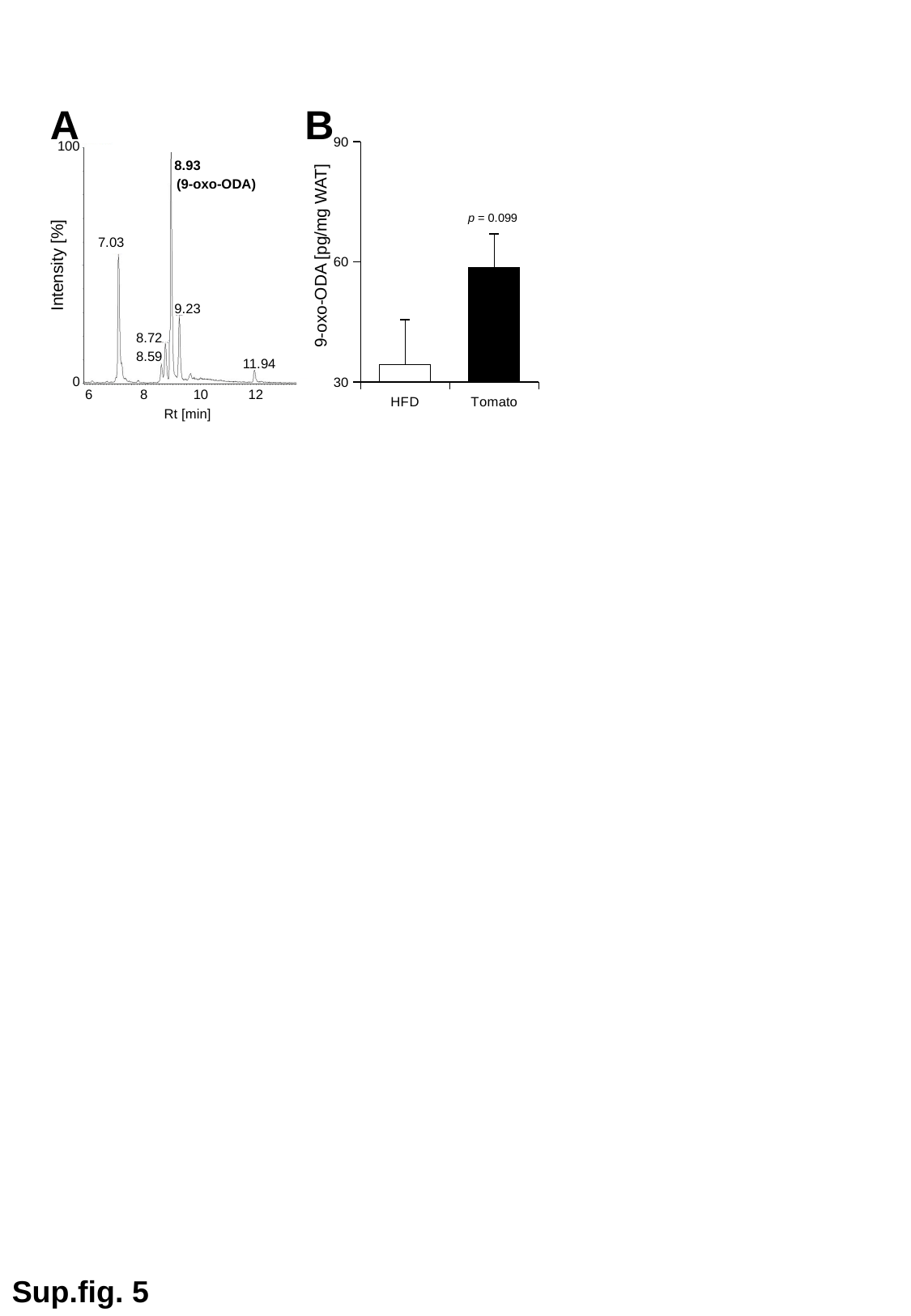

A
B
100
### Chart
| Category | |
|---|---|
| HFD | 34.41477638613745 |
| Tomato | 58.607208345654556 |8.93
(9-oxo-ODA)
7.03
9-oxo-ODA [pg/mg WAT]
Intensity [%]
9.23
8.72
8.59
11.94
0
6
8
10
12
Rt [min]
Sup.fig. 5

Supplement: S5 Fig — (A) The extracted ion chromatogram (m/z = 293.209) in tomato extract sample. (B) The amount of 9-oxo-ODA in white adipose tissue. Data are presented as means ± SEM (n = 8–10/group). (PPTX) [file pone.0191203.s005.pptx]
