# Supplementary material for: Wide-range screening of anti-inflammatory compounds in tomato using LC-MS and elucidating the mechanism of their functions
Source: PLoS One. 2018 Jan 12;13(1):e0191203. doi: 10.1371/journal.pone.0191203 (PMC5766234; doi:10.1371/journal.pone.0191203)
Supplement: S2 Fig — RAW264.7 cells were stimulated with LPS (100 ng/mL) and incubated with tomato extract for 1h. Total cell lysates were extracted from cultured RAW264.7 cells. The quantification of western blot signals on (A) JNK1, (B) JNK2/3, (C) ERK1, (D) ERK2, (E) p38 phosphorylation, and (F) IκB-α degradation. Data are presented as means ± SEM (n = 3–4/group). *p < 0.05, **p < 0.01 vs. culture treated with LPS alone. (PPTX) [file pone.0191203.s002.pptx]

## Slide 1
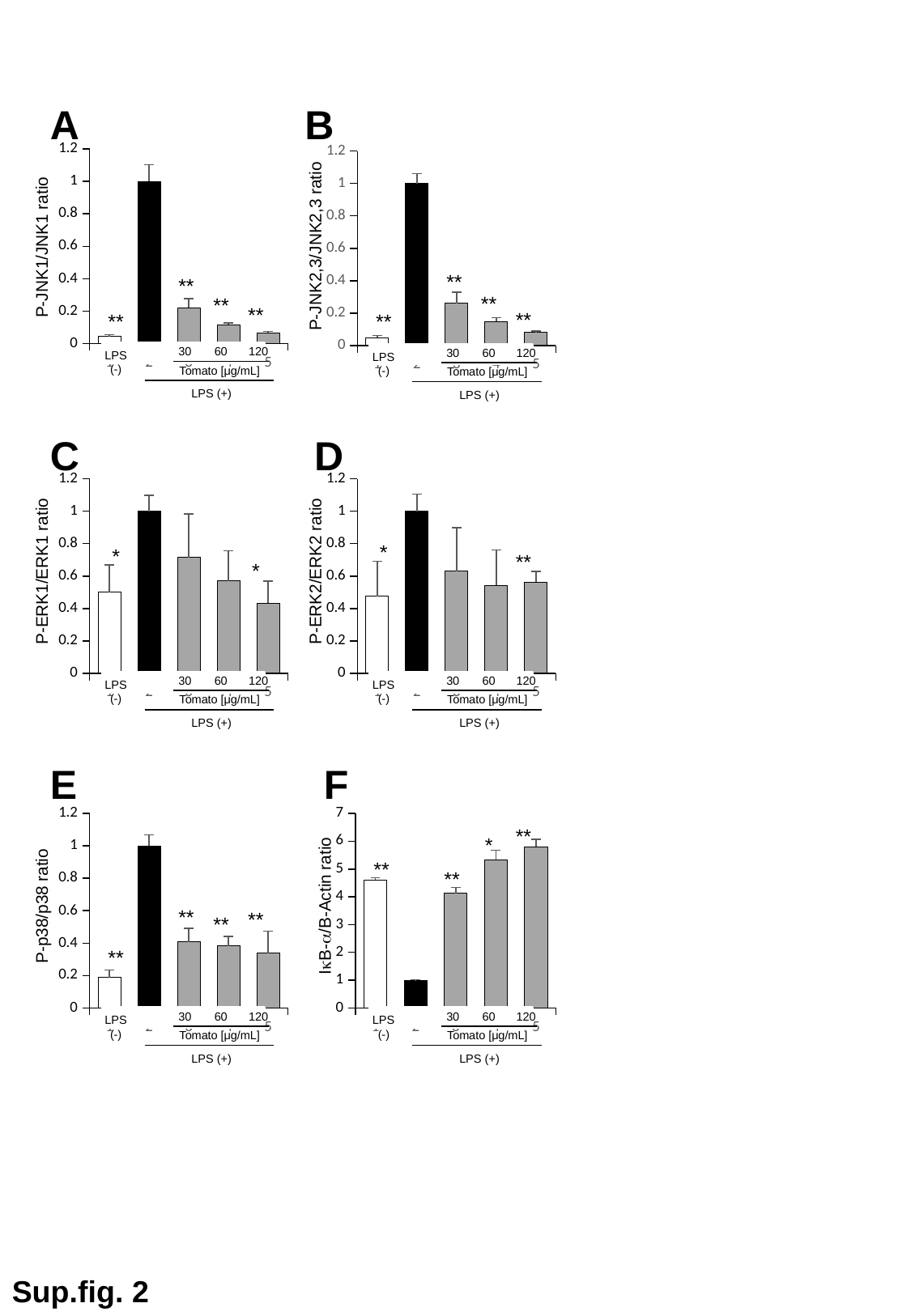

A
B
### Chart
| Category | |
|---|---|
### Chart
| Category | |
|---|---|P-JNK2,3/JNK2,3 ratio
P-JNK1/JNK1 ratio
**
**
**
**
**
**
**
**
30
60
120
LPS
(-)
Tomato [μg/mL]
LPS (+)
30
60
120
LPS
(-)
Tomato [μg/mL]
LPS (+)
C
D
### Chart
| Category | |
|---|---|
### Chart
| Category | |
|---|---|*
*
**
*
P-ERK1/ERK1 ratio
P-ERK2/ERK2 ratio
30
60
120
LPS
(-)
Tomato [μg/mL]
LPS (+)
30
60
120
LPS
(-)
Tomato [μg/mL]
LPS (+)
E
F
### Chart
| Category | |
|---|---|
### Chart
| Category | |
|---|---|**
*
**
**
P-p38/p38 ratio
IkB-a/B-Actin ratio
**
**
**
**
30
60
120
LPS
(-)
Tomato [μg/mL]
LPS (+)
30
60
120
LPS
(-)
Tomato [μg/mL]
LPS (+)
Sup.fig. 2
